# In-depth study on the effect of oxygen-containing functional groups in pyrolysis oil by P-31 NMR†

**DOI:** 10.1039/c9ra04099d

**Published:** 2019-08-29

**Authors:** Zhihong Wu, Haoxi Ben, Yunyi Yang, Ying Luo, Kai Nie, Wei Jiang, Guangting Han

**Affiliations:** Southeast University Nanjing 210096 China benhaoxi@gmail.com; Key Laboratory of Energy Thermal Conversion and Control of Ministry of Education, School of Energy and Environment Nanjing 210096 China; Qingdao University Qingdao 266071 China; State Key Laboratory of Bio-Fibers and Eco-Textiles, Qingdao University Qingdao 266071 China

## Abstract

One of the major obstacles to the widespread use of pyrolysis oil is its high oxygen content, with oxygen atoms being mainly present in the hydroxyl and the carboxyl groups. Therefore, quantitative and accurate characterization of oxygen-containing functional groups is of great significance. This study employed ^31^P NMR to conduct in-depth studies on several model compounds including four kinds of alcohols and carboxylic acids. The model compounds have been investigated for stability in ^31^P NMR solution for both short storage (4 hours) and long storage (14 days), namely by *in* and *ex situ* monitoring. The experimental phenomena indicates that carboxylic hydroxyl has poor stability compared to alcohols hydroxyl group, which is reflected in the amount of alcohol compounds remaining over 90% after long-term storage. Among the carboxylic acids used in the study, aromatic acids are relatively stable. Interestingly, oxalic acid is extremely unstable and completely decomposed in the first hour, while formic acid had only a small amount left after one day of storage. Therefore, the optimum time for the preparation, storage and upgrading of the pyrolysis oil can be determined by analysis of the stability of the oxygen-containing functional groups in ^31^P NMR solution to ensure accuracy. Moreover, according to the results of the ^31^P NMR and other characterization methods, it can been seen that water was formed during the decomposition of all the model compounds. This is a report on the quantitative characterization of different oxygen-containing functional groups representing pyrolysis oil and the first study on the similarities and differences of the decomposition of carboxylic acids and alcohols in ^31^P NMR solution. The results of this in-depth investigation can provide important assistance in research that will further upgrade and apply pyrolysis oil.

## Abstract 2

Both aliphatic and carboxylic OH undergo the same decomposition pathway to form water during *in situ*^31^P NMR monitoring.

## Graphical abstract

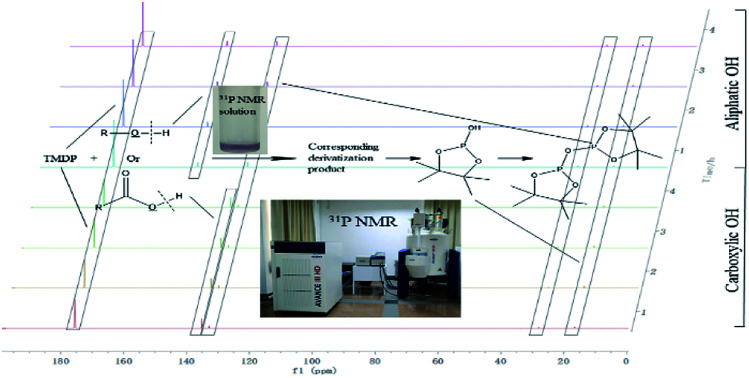

## 1. Introduction

In the last decade, research on biomass conversion has attracted considerable attention, mainly due to the consumption of fossil energy.^1^ In addition, the resulting growing greenhouse gas emissions has sparked interests in the search for alternative energy.^2^ Among the various alternative energy sources, biomass-converted fuels are characterized by carbon neutrality and large aggregates, and are therefore expected to ease the pressure on energy supply.^3–5^ At present, researchers in this field worldwide have paid more attention to various biomass conversion methods, such as gasification and pyrolysis, which have been developed.^6^ Pyrolysis stands out among the various conversion technologies that have been studied, driven by low economic investment costs, which is considered to be one of the most promising methods for producing biomass-derived conversion oil and biomaterials.^7^ However, several challenging properties, namely acidity, corrosiveness, poor volatility, aging problems and low heating value, have hampered the production of bio-oils for use as intended fuels.^5,8^ High oxygen content caused by oxygen atoms presented in oxygen-containing functional groups is contributed to the major drawback of pyrolysis bio-oils.^9^ Therefore, the subsequent upgrade process is critical in reducing the oxygen content of the biomass-derived intermediates for conversion into a renewable fuel that can be used directly.^10^ Further quantitative, precise and in-depth characterization of oxygen-containing functional groups in biomass-converted fuels is an indispensable step for its commercial applications. It has been reported that some traditional analytical methods, such as Fourier transform infrared spectroscopy (FI-IR), Gas Chromatography-Mass Spectrometer (GC-MS), and Gel Permeation Chromatography (GPC), always be used to investigate individual components in pyrolytic oil.^11–13^ However, the complexity of pyrolysis products brings huge obstacles to these commonly used analysis methods. In earlier studies, GC-MS was frequently utilized by researchers to determine the composition of pyrolysis liquid products, which only identified about 40% portion caused by the poor volatility.^14,15^ To overcome the limitation of GC-MS, FT-IR is highly anticipated to analyze the whole components in pyrolysis oil,^16–18^ but, its application is hindered by the inability to achieve quantitative analysis.^19^ In addition to these methods, GPC has the ability to obtain the distribution of molecular weight of compounds since this parameter is an important factor determining the physical properties.^20^ However, such an analysis result is not sufficient to explore the upgrading process of biomass-derived oil.^21^ Therefore, the achievement of accurate and quantitative characterization of pyrolysis oil is of great significance for studying the mechanism of thermochemical decomposition and upgrading processes.

In addition to the characterization methods mentioned above, nuclear magnetic resonance (NMR) has the ability to obtaining detailed structural information and has been gradually applied to the study of biomass thermochemical conversion.^22^ Dissolving bio-oils with a suitable solvent to obtain presenting functional groups' information becomes the main competitiveness of this method, which is independent of volatility.^23^ Various NMR methods has be employed to characterize pyrolysis oil, which has reinvigorated interest among researchers. Schnitzer *et al.* employed ^1^H NMR to determine the composition of the product of fast pyrolysis of chicken manure.^24^ In addition, quantitative ^1^H NMR characterization was used in Boscagli *et al.* for the hydrotreating of bio-oils prepared from straw.^25^ The data obtained based on such a method confirmed the results obtained by GC-MS, indicating that the conversion of phenol and the selectivity of the product were all different. In their subsequent study, characterization was performed by techniques such as ^1^H NMR to compare the activity of nickel-based catalysts for catalytic pyrolysis of oils and model compounds for hydrodeoxygenation processes.^26^ In the study conducted by Strahan *et al.*, chemical functional group composition in pyrolysis oil can be better determined by using ^13^C NMR than ^1^H NMR.^27^ However, the application of these commonly used one-dimensional (1-D) NMR characterization techniques is limited by spectral overlap problems.^27^ The two-dimensional NMR has emerged as a technique for effectively reducing above phenomena.^9^ Heteronuclear multiple quantum correlation (HSQC) NMR as such a method can provide a complete analysis of bio-oils, and the chemical shift assignments of variety kinds of C–H bond contained in the biomass-converted oil.^9^ In Ben's study, the detailed characterization of pyrolysis oil obtained from tannin and hemicellulose were accomplished using HSQC-NMR.^28^ Nevertheless, the above techniques are still not quantitative enough and spectral overlap still exists.^29^

**Fig. 1:**
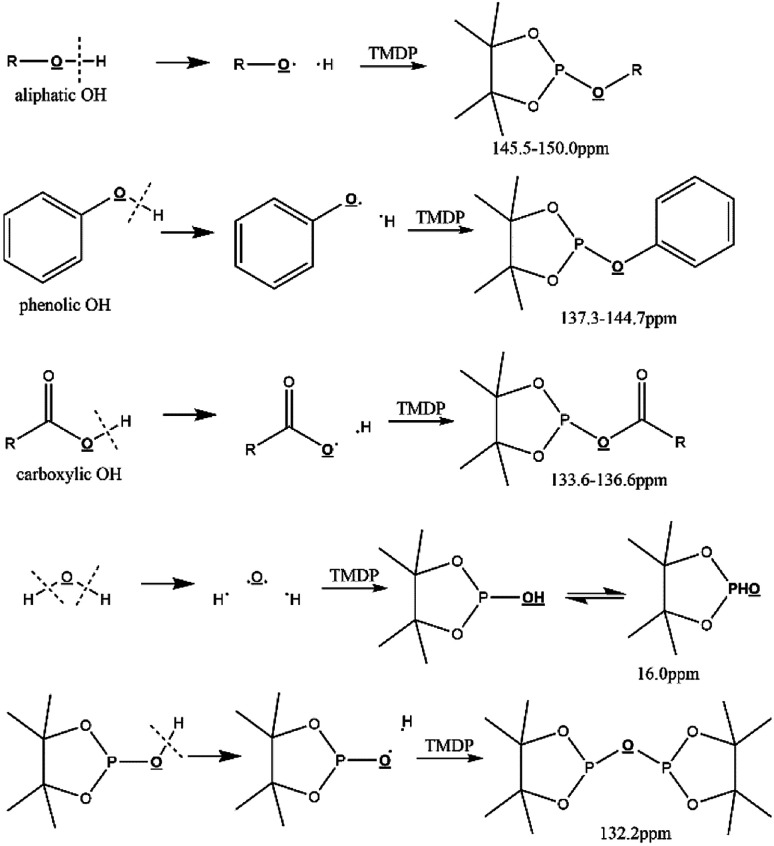
Reactions of the phosphorous reagent (TMDP) with various hydroxyl functional groups and the ^31^P NMR assignment of phosphitylated compounds (reproduced from ref. 32 with permission from the Royal Society of Chemistry).^32^

Due to 100% nature abundance of phosphorus-31 nucleus, ^31^P NMR can achieve high sensitivity during NMR experiments, further making up for the overlap of ^1^H NMR and unsatisfactory relaxation time problem of ^13^C NMR.^24^ The utilize of ^31^P NMR to detect and analyze hydroxyl groups has been around for several decades.^30^ 2-chloro-4,4,5,5-tetramethyl-1,3,2-dioxaphospholane (TMDP), a commonly used phosphorylating reagent, can be derivatized with different hydroxyl-containing compounds to form products that can generate signal in ^31^P NMR, which is shown in Fig. 1.^31,32^ In addition, it has been experimentally found that triphenylphosphine oxide (TPPO) is identified as the best internal standard for the ^31^P NMR characterization because of its good stability.^32^ The chemical shifts and integral regions of different hydroxyl groups derived from TMDP have been studied and presented in Table S1.†^32^

One of the earliest reports on the application of ^31^P NMR to characterize hydroxyl functional groups is an analysis of coal pyrolysis condensates and extracts completed by Wroblewski's research groups.^33^ Subsequently, ^31^P NMR has been widely used as an effective quantitative characterization method for decades. In addition, Schiff *et al.* found in the experiment that the quantitative information of the product derived from hydroxyl-functional compound and a phosphorylating reagent can be determined through ^31^P NMR, and corresponding chemical shift of phosphorus in the derivatization product is significantly different.^34^ It has been reported that an unprecedented analytical method-quantitative ^31^P NMR was used to quantitatively characterize all functional groups by labelling of all unstable hydroxyls with a phosphorus-containing reagent.^35^ A study of the Li Mi's research team on the chemical shifts of phosphorylated products from 54 different compounds and TMDP in ^31^P NMR can provides valuable and precise spectral information for characterizing lignocellulose-based compounds.^36^ The heavy oil obtained from the pyrolysis of pine trees after automatic hydrolysis was studied by employing quantitative ^31^P NMR, which found that the yield of levoglucosan in bio-oil was increased by using this pre-treatment method.^37^ Autohydrolysis is a pretreatment method that addresses the unfavorable properties of pyrolysis oils, producing acetic acid from hemicellulose acetyl groups using elevated temperatures (140–220 °C) without the use of chemical additives.^37^ More recently, a novel method for *in situ* monitoring of ^31^P NMR is applied to detect time-changes of pyrolysis oil in corresponding solution.^32^ This is the first time that such a unique method has been devoted to determine the stability of biomass-converted oil. The same monitoring method was used in his other study to determine the change in aliphatic OH bond of pine pyrolysis oil after zeolite treatment.^38^ Biomass thermochemical converted oil undergone the significant deoxygenation reaction during the upgrading process accompanied by a decrease in oxygen-containing groups, which can be detected by mentioned method and consistent with other characterization methods. Therefore, this analytical method has attracted a wide range of interest in charactering oxygen-containing functional groups pyrolysis oil.^31^

One of the major obstacles to the widespread use of pyrolysis oil is the high oxygen content, while oxygen atoms are mainly present in the hydroxyl and the carboxyl. Therefore, quantitative and accurate characterization of oxygen-containing functional groups is of great significance for reducing the oxygen content of pyrolysis oil, namely the upgrading process. The main purpose of this paper is to obtain precise and quantitative data for the analysis of several different hydroxyl groups that represent pyrolysis oils using a novel characterization method. Several carboxylic acid and alcohol model compounds, namely aliphatic, benzoic acid, and methanol, ethylene glycol, 1,2-propanediol, and glycerin have been studied for their stability in ^31^P NMR by both *in situ* and *ex situ* monitoring. Such a monitoring method can provide powerful assistance in providing accurate and quantitative characterization data. A few years ago, Ben *et al.* used this method to monitor the change of pyrolysis oil over time and found that many of the functional groups were unstable.^32^ In their other study, the same method was used to explore the upgrade process for some ring opening intermediates.^10^ In addition, Alistaur *et al.* used ^31^P NMR to determine the solubility of lignin phenols and wood in the imidazole chloride *in situ*, which provides additional information on the structure and solubility of natural wood biopolymers.^39^ Ragauskas *et al.* used *in situ* NMR characterization to explore the chemical structure changes of pyrolysis oil during aging, which provides insights into the mechanism of the aging process.^12^ In this study, corresponding data shows that all compounds used for this study change more or less during the monitoring period. This is a report on the quantitative characterization of different oxygen-containing functional groups representing pyrolysis oil and the first study on the similarities and differences of the decomposition of carboxylic acids and alcohols in ^31^P NMR solutions. The results of this in-depth investigation can provide important assistance in the study of the upgrading of pyrolysis oil and its application.

## 2. Materials and methods

The required chemicals in the experiments, including the model compounds studied, were obtained from the Tansoole (the official website of Shanghai Titan Technology Co., Ltd., located in Shanghai, China) and used immediately upon receipt. All NMR spectra obtained in the experiment were recorded with a Bruker Avance/DMX 400 MHz NMR spectrometer and processed by using MestReNova-11.0.4 software default processing template including baseline correction and automatic phase adjustment.

### 2.1 Sample preparation

The high oxygen content properties hinder the industrial application of pyrolysis oil. Moreover, the problem of corrosion and aging of pyrolysis oil is also due to the presence of oxygenates such as carboxylic acids. Therefore, research on different oxygen-containing functional groups is an indispensable step in exploring biomass conversion liquid products. However, due to the complexity of biomass conversion products, study at the molecular scale is of great significance for exploring mechanism. In this study, some model compounds containing different hydroxyl groups representing pyrolysis oil were selected for experiments, namely formic acid, acetic acid, oxalic acid, benzoic acid, and methanol, ethylene glycol, 1,2-propanediol, and glycerin. Firstly, 2.31 ml of CDCl_3_ was mixed with 6.3 mg of a relaxing reagent (Cr(acac)_3_) and 41.7 mg of an internal standard (TPPO) as an original solution. Then, anhydrous pyridine with a volume ratio of CDCl_3_ of 1.6 : 1 was added and mixed with the original solution in a scintillation vial. Subsequently, about 0.5 ml of the solution was taken in the vial for each experiment, and about 0.1 ml of TMDP and one drop of the model compound were added. After shaking well and ensuring no precipitation, the liquid was transferred into an appropriate NMR tube with nitrogen purge. The weights of different model compounds, original solution, anhydrous pyridine, and TMDP are summarized in Table S2.†

### 2.2 Quantitative method of ^31^P NMR characterization

^31^P NMR spectral data was obtained by typing “zgig” using a reverse gated decoupling pulse sequence of 90% pulse angle, and other parameters are shown in Table S3.†^32^ In addition, in order to ensure that the parameters used in the experiment meet the quantitative requirements, the methanol sample was subjected to *in situ*^31^P NMR monitoring using different *D*_1_ time, and the results are shown in Fig. S1 and Table S4.† It has been reported^40^ that *T*_1_ decreases with increasing molecular weight, while methanol has the lowest molecular weight among all the model compounds used. The results demonstrate that the parameters in this study are available.

For the monitoring process, whether *in situ* or *ex situ*, a nitrogen purge was performed to isolate oxygen and water vapor, which avoided the effects of other side reactions, such as oxidation reactions. For short real-time monitoring, the samples were tested continuously for four hours to ensure that the reaction was monitored in real time. By carrying out such a method, changes in the derivatization of different oxygen-containing functional groups in ^31^P NMR solution were recorded, which were used to determine the real-time changes in the reaction of all model compounds. *In situ* NMR monitoring can provide strong evidence for studying the mechanism of change during a short-term (4 hour) reaction. In addition, *ex situ* monitoring is the detection of a reaction after a certain period of time, with more attention to product information. In this study, the *ex situ* monitoring was performed once after 1 day, 7 days, and 14 days later, which can obtain information on the products that the derivatized compounds would produce in ^31^P NMR solution after these times. *In situ* monitoring was concerned with the first 4 hours of change in the derivatization reaction, while *ex situ* monitoring was more committed to the results of reactions after a certain time point (1 day, 7 days, 14 days).

### 2.3 Qualitative method of ^1^H NMR characterization

The parameter of qualitative ^1^H NMR was set as delay time of 8 s and scanning times of 16 with more parameters shown in Table S3.† At room temperature, the spectra were obtained by entering the command “zg” to start the scan.

### 2.4 Qualitative method of ^13^C NMR characterization

A spectrum of ^13^C NMR was shown by entering a common pulse sequence (zgig) with a relaxation delay of 1.5 s. In addition, the number of scans is set to 1500 with other testing parameters shown in Table S3,† which is done at room temperature.

## 3. Results and discussion

### 3.1 Determination of the stability of several carboxylic acids in ^31^P NMR solution

**Fig. 2:**
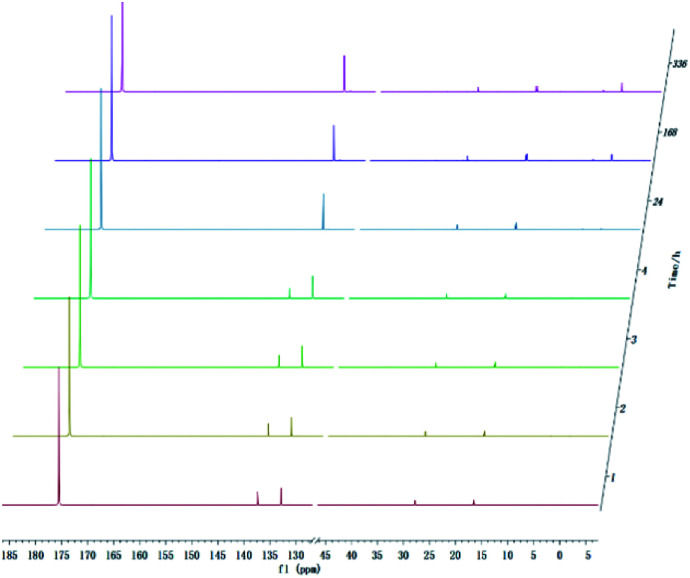
*In situ* and *ex situ* monitoring for the stability of formic acid during ^31^P NMR with TPPO as internal standard.

With development for decades, ^31^P NMR has been widely devoted to quantitatively characterize biomass conversion products such as pyrolysis oil. In the previous reports, acid groups in the produced bio-oil have been detailed to cause aging, corrosion and other problems.^12^ Therefore, further quantitative and accurate characterization of several carboxyl acids is an indispensable step for biomass-converted oil's commercial applications. The short-term real-time monitoring of several carboxyl-containing model compounds including aliphatic acids and benzoic acid were first accomplished in this study, as well as *ex situ* experiments. The corresponding chemical shifts and integration regions for different products derived from model compounds and TMDP measured by quantitative ^31^P NMR are shown in Table S1† and naming of some compounds mentioned in this study is shown in Table S5.† In order to avoid oxidation reaction, the NMR tube was filled with nitrogen. The results of the stability test of formic acid are shown in Fig. 2 and Table 1, which indicates formic acid was unstable, decreased dramatically in a day during the monitoring period. Moreover, it is interesting to find that this model compound was almost completely decomposed after long-term storage. These results can also been seen in Table 1. From the result of integral calculation can be shown, the amount of carboxyl groups was reduced by ∼30% after 4 hours and by ∼99% after 14 days. Since no precipitate formation was observed in the experiment, the possible cause of such a larger reduction in formic acid in the sample was decomposition to form new compounds. Several new peaks located at ∼0.2 ppm and ∼3.7 ppm (^31^P NMR) were formed and gradually grown during the monitoring period. In addition, some small peaks in aliphatic area with the range of ∼145.5 to ∼150.0 ppm (^31^P NMR) appeared and increased slightly. It indicates that aliphatic hydroxyl products might be produced after monitoring process. In addition, as can be seen from the results, TMDP was continuously consumed over time, which indicates that the decomposition products of formic acid would continue to perform derivative reaction with TMDP. Furthermore, the two water peaks (∼16.0 ppm and ∼133.2 ppm (^31^P NMR), representing the compounds derived from TMDP and water) show a trend of gradual rise (Fig. 2). During the *ex situ* monitoring process, the water content in the sample increased from the initial 2.35 wt% to about 4 wt%, which is shown in Table 1. This change in water content indicates water was formed as the decomposition progressed. The monitoring results of formic acid for both short and long time are consistent with Ben's analysis.^32^ Other analytical techniques such as ^1^H and ^13^C NMR were used to demonstrate the results of this chemical in ^31^P NMR solution by *in situ* monitoring.

**Table 1:** Different hydroxyl group contents and water wt% for the formic acid during 4 hours of *in situ* and 14 days of *ex situ* stability tests with TPPO as internal standard

Time	Aliphatic OH^a^ mmol g^−1^	Phenolic OH^a^ mmol g^−1^	Carboxylic OH^a^ mmol g^−1^	H_2_O^b^ wt%
1 h	0.0000	0.0001	0.0434	2.35
2 h	0.0001	0.0006	0.0406	2.54
3 h	0.0002	0.0012	0.0368	2.73
4 h	0.0004	0.0002	0.0325	2.77
1 d	0.0002	0.0004	0.0016	3.82
7 d	0.0002	0.0004	0.0007	4.01
14 d	0.0004	0.0008	0.0004	3.89

a The calculation method: (integration of spectral region of interest × mmol TPPO)/(integration of TPPO region × total mass (g) of NMR sample).

b The percentage calculation method: (integration of water region × mmol TPPO × 278.29 g mol^−1^ TPPO × 100)/(1000 × TPPO purity × integration of TPPO region × total mass (g) of NMR sample).

As shown in the spectrum of ^1^H NMR (Fig. S2†) during the *in situ* monitoring period, the peak of product formed from formic acid reacted with TMDP (∼8.33 ppm, hydrogen proton in HC=O) gradually decreased and almost disappeared after long-term storage (shown in Fig. S3†), which agrees with the result in ^31^P NMR (Fig. 2). In addition, the *in situ*^13^C NMR (Fig. S4†) also shows a falling peak located at ∼160.6 ppm representing compound 1 (Fig. S5†). All of these changes in the three spectra indicate that formic acid is extremely unstable and can be completely decomposed. Moreover, there was a slight growing peaks located at ∼6.4 ppm in ^1^H NMR (Fig. S2†). Correspondingly, such rising peak also exist in ^13^C NMR (Fig. S4†), which is ∼85.5 ppm. In Table 1, it can be seen that aliphatic hydroxyl group increased gradually, and there was a small amount of aliphatic peaks formation in the ^31^P NMR (Fig. 2). As formic acid decreased, aliphatic product (compound 4 in Fig. S5†) was produced during the monitoring period. In summary, two decomposition pathways of formic acid are proposed in Fig. S5.† These conclusions are consistent with the findings of Ben.^32^

**Fig. 3:**
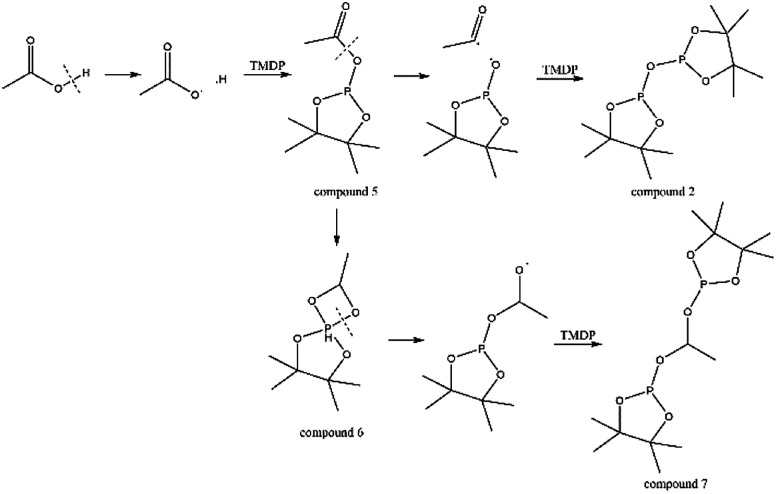
Tentative decomposition pathway for acetic acid in ^31^P NMR.

**Fig. 4:**
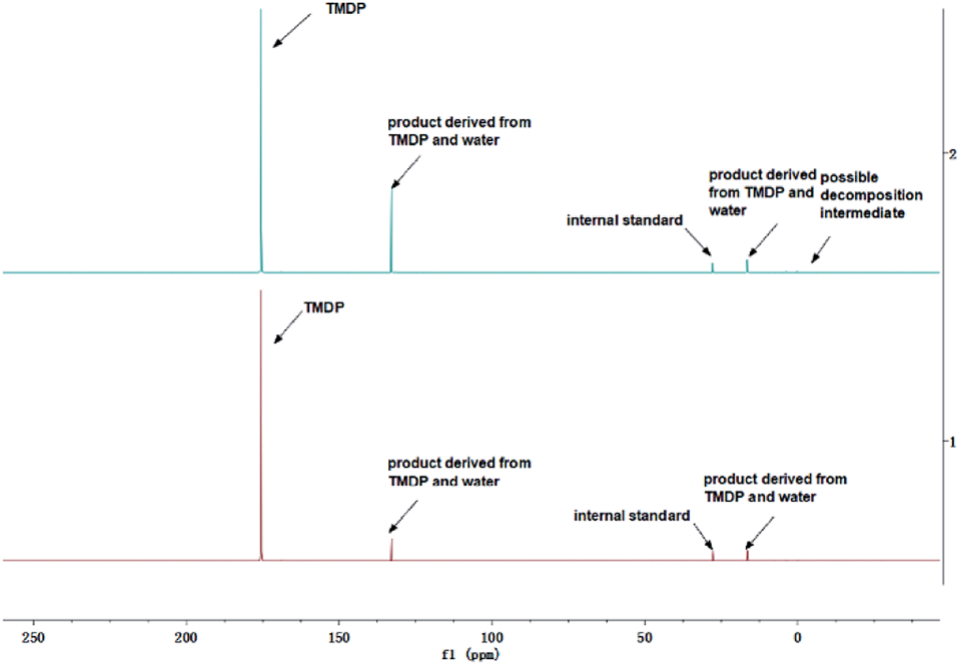
Comparison before (bottom) and after (top) adding oxalic acid to the solution in the ^31^P NMR.

**Fig. 5:**
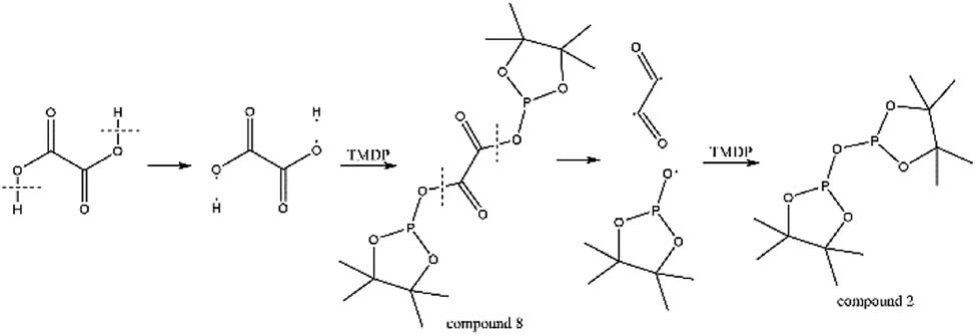
The major decomposition pathway for oxalic acid in ^31^P NMR.

The *in situ* monitoring ^31^P, ^1^H and ^13^C NMR for the stability of acetic acid were also accomplished during this study. The spectrum of ^31^P NMR and integration result are shown in Fig. S6 and Table S6.† The peak located at ∼132.3 ppm in ^31^P NMR decreased over time, indicating that the content of acetic acid in ^31^P NMR solution was gradually reduced, which is consistent with the result in Table S6.† Compared to formic acid, acetic acid was relatively stable during the monitoring process. Even so, it has been decreased by nearly 40% after being placed for a long time (14 days). However, the optimum time for testing and preparation can be determined from this result. Several new peaks located at ∼20.9 ppm, ∼33.5 ppm and ∼147.1 ppm were formed and gradually grown during the *ex situ* monitoring period in Fig. S6† (^31^P NMR), which shows that acetic acid was decomposed into more and complex products in comparison to formic acid. The water content in the solution increased to 4.17 wt% during the *in situ* monitoring period and conversely, decreased after long-term storage. Therefore, water is one of the major decomposition products, followed by reactions with other products to form new compounds. As the results shown in Table S6,† the contents of hydroxyl groups in aliphatic compounds increased during this study, which shows that both types of compounds were also contained in the acetic acid decomposition products. Further investigation on ^1^H and ^13^C NMR of this model compound has been completed. The peaks at ∼22.6 ppm, ∼174.6 ppm (^13^C NMR, Fig. S7†) and ∼1.98 ppm (^1^H NMR, Fig. S8†) gradually decreased, which supports the result of ^31^P NMR. It indicates that corresponding compound 5 (in Fig. 3) formed by reaction between acetic acid and TMDP was unstable. Several new peaks formed at ∼24.6 ppm and ∼91.2 ppm has been shown in the *ex situ* monitoring ^13^C NMR (Fig. S9†), which belongs to the peak of compound 2 (Fig. 3). The *ex situ* monitoring ^1^H NMR results also indicate that the compound 7 was formed during the acetic acid decomposition process, which is represented by peak formation at ∼2.25 ppm in Fig. S10.† In addition, there was a growing peak located at ∼24 ppm in ^13^C NMR. Correspondingly, a similar peak at ∼1.45 ppm (Fig. S11†) was gradually growing during the *in situ* monitoring in the ^1^H NMR, which may represent possible decomposition products other than compound 7 in Fig. 3. In summary, the tentative decomposition pathway of acetic acid in ^31^P NMR solution is shown in Fig. 3. In this study, interestingly, there is no peak of the product from oxalic acid and TMDP during the first hour of monitoring in ^31^P NMR (the spectrum in Fig. 4). In comparison with the results when no oxalic acid was added, it is interesting to found that the peak intensity located at ∼16.7 ppm and ∼132.9 ppm (^31^P NMR) representing the products from water and TMDP is significantly increased, which is shown in Fig. 4. The results in Table 2 also indicate that the content of the product from water and TMDP (compound 2 in Fig. 5) increased dramatically. Therefore, in the decomposition of this dibasic acid, water is produced in large quantities, which is consistent with the results of aliphatic acid analyzed above. Possible reason for the rapid decomposition of the product derived from oxalic acid and TMDP may be the presence of two carboxyl functional groups in oxalic acid, resulting in a large increase in water content. The possible decomposition pathway for oxalic acid in the state studied has been assumed (Fig. 5).

**Table 2:** Comparison before (bottom) and after (top) adding oxalic acid to the solution on different hydroxyl group contents and water wt%

Sample	Aliphatic OH^a^ mmol g^−1^	Phenolic OH^a^ mmol g^−1^	Carboxylic OH^a^ mmol g^−1^	H_2_O^b^ wt%
No oxalic acid	0.0000	0.0000	0.0000	1.98
Oxalic acid	0.0002	0.0004	0.0001	5.43

a The calculation method: (integration of spectral region of interest × mmol TPPO)/(integration of TPPO region × total mass (g) of NMR sample).

b The percentage calculation method: (integration of water region × mmol TPPO × 278.29 g mol^−1^ TPPO × 100)/(1000 × TPPO purity × integration of TPPO region × total mass (g) of NMR sample).

In addition to these aliphatic model compounds, one of the aromatic acids has been studied with its spectrum shown in Fig. S12.† However, it is shown from the figure that the content of benzoic acid changed slightly compared to other model compounds mentioned above. The results of different OH groups' contents of the benzoic acid during the stability tests are shown in Table S7,† as well as water, which also indicate that the product from benzoic acid and TMDP decomposed slowly with a 17.37% reduction during *ex situ* monitoring process. In addition, there were some new peaks formed located at ∼0.3 ppm, ∼0.5 ppm and 138.3 ppm in ^31^P NMR, which may due to the formation of new compounds. In contrast, there were some slightly growing peaks located at ∼16.5 ppm and ∼132.9 ppm in Fig. S12,† which represents the compound derived from water and TMDP. The data in Table S7† also indicates that the water content is gradually increasing during the monitoring period. As a result above, compound nine (derived from TMDP and benzoic acid, in Fig. S13†) would gradually decompose into compound 2. Further study on *in situ* monitoring ^1^H and ^13^C NMR for benzoic acid in ^31^P NMR solution have been accomplished. The *in situ*^13^C NMR (Fig. S14†) shows that the product formed after TMDP reacted with benzoic acid and is variable and can be gradually decomposed. In addition, the *in situ*^1^H NMR results (Fig. S15†) also indicate that the hydrogen atom on the benzene ring of compound 9 (Fig. S13†) gradually decreased during the monitoring period. All of these phenomena indicates that benzoic acid gradually decomposed in ^31^P NMR solution and generated water. Based on the above analysis, a decomposition process diagram of benzoic acid can be obtained and shown in Fig. S13.†

### 3.2 Determination of the stability of several alcohols in ^31^P NMR solution

**Fig. 6:**
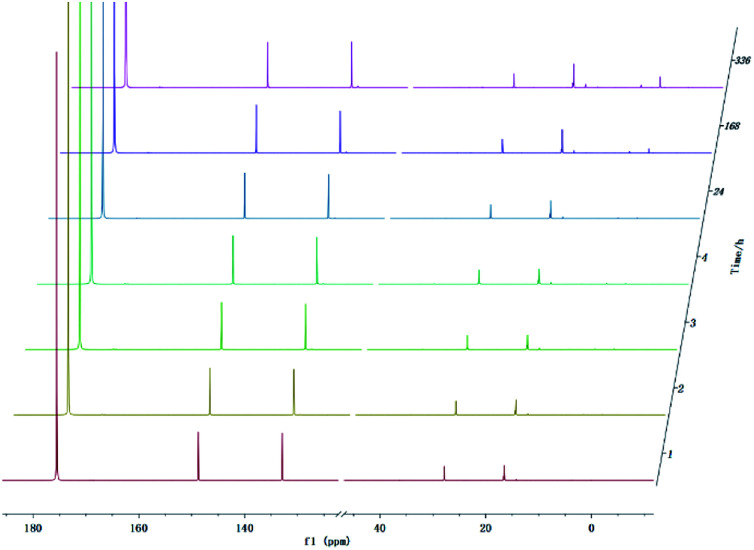
*In situ* and *ex situ* monitoring for the stability of methanol during ^31^P NMR with TPPO as internal standard.

In addition to being present in the carboxyl group, the oxygen atom also constitutes hydroxyl group presenting in the pyrolysis oil. Therefore, the precise and quantitative characterization of different hydroxyl-functional compounds is of great importance for upgrading pyrolysis oils. Several alcohol model compounds have been investigated on the stability in ^31^P NMR. The in and *ex situ* monitoring ^31^P NMR results (Fig. 6) show that there is a small change in the peak of the product from methanol and TMDP during the monitoring period. According to the integral results in Table S8,† the amount of OH groups in aliphatic compounds decreased by ∼1.68% after 4 hours and by ∼2.56% after 14 days, which indicates that methanol is relatively stable compared with carboxylic acids. Since no precipitation occurred during the monitoring process, the cause of this phenomenon was the gradual decomposition of methanol. In contrast, the content of the compound derived from water and TMDP increased from the initial 2.05% to 2.56% after long-term storage. Therefore, water accounts for a large proportion of methanol decomposition products. Similarly, a new peaks formed can been seen in Fig. 6 and gradually grown located at ∼33 ppm (^31^P NMR). In addition, *in situ* monitoring process of the derivatization between TMDP and methanol by both ^1^H NMR and ^13^C NMR. The spectrum of ^13^C NMR (Fig. S16†) shows that the peak representing compound 10 (Fig. S18†) was slightly decreased during *in situ* monitoring period. Similarly, the *in situ*^1^H NMR (Fig. S17†) results also indicate that the product derived from methanol and TMDP can gradually decompose over time, which is consistent with the results of ^31^P NMR. Unlike carboxylic acid compounds, methanol is relatively stable and is mainly decomposed to form water. The possible decomposition pathway is shown in Fig. S18.†

In addition to methanol, several polyol model compounds have also been studied. From the ^31^P NMR spectrum of ethylene glycol (Fig. S19†), the peak of the product from it and TMDP is slightly reduced during the monitoring process. The integration results (Table S9†) also show a decrease in aliphatic OH content from 0.1283 mmol g^−1^ to 0.1230 mmol g^−1^ after 14 days, which change is not large relative to carboxylic acids. Conversely, the water content increased by 0.3% after long-term storage, which means that water is produced during the decomposition of the product from ethylene glycol and TMDP. In addition, there were some gradually decreasing peaks located at ∼63.5 ppm and ∼84.9 ppm representing the bond in compound 11 (Fig. S20†) in ^13^C NMR spectrum (Fig. S21†). Correspondingly, there was also a rising peak in Fig. S22,† which supports the results of ^31^P NMR. A hypothetical decomposition path has been proposed in Fig. S20,† which is based on the above analysis. In addition, experiments on another polyol model compound 1,2-propanediol have also been completed. Like other alcohols, the reaction product of 1,2-propanediol and TMDP was relatively stable and decreased slowly, which is shown in Fig. S23.† The results in Table S10† also indicate that the compound 12 (Fig. S24†) content was reduced by 5.9% during the *ex situ* monitoring period. With the decrease of 1,2-propanediol, the water content (both peaks at ∼16.5 ppm and ∼132.9 ppm in ^31^P NMR) increased gradually. Therefore, water is one of the main product of 1,2-propanediol decomposition. As shown in the spectrum of *in situ* monitoring of ^13^C NMR (Fig. S25†), the peaks located at ∼22 ppm, ∼68 ppm and ∼85 ppm representing the carbon in compound 10 (Fig. S24†) gradually decreased during the *in situ* monitoring process. Moreover, the ^1^H NMR results (Fig. S26†) indicate that 1,2-propanediol (both peaks at ∼3.75 ppm and ∼4.35 ppm, which are the product from 1,2-propanediol and TMDP) decomposed slowly. In summary, the main decomposition pathway of 1,2-propanediol is the formation of water, which is shown in Fig. S24.† For glycerol, it decomposed slowly, which show as a decreasing peak located at ∼147.5 ppm in Fig. S27† (the spectrum of ^31^P NMR). The results in Table S11† show that the content of aliphatic OH changed from 0.1770 mmol g^−1^ to 0.1696 mmol g^−1^, which is consistent with the variation trend in Fig. S27.† On the contrary, the content of water in ^31^P NMR solution increased by 0.1% (Table S11†), which is reflected in Fig. S27† that the peaks at ∼16.1 ppm and ∼133.1 ppm in ^31^P NMR show an upward trend. The possible cause of this change is the decomposition of glycerol into water. Therefore, a convincing reaction way has been shown in Fig. S28.† Further ^13^C NMR studies showed compound 13 (Fig. S28†) can be gradually decomposed, which is manifested by a decrease in its peaks (both peaks located at ∼63.8 ppm and ∼73.2 ppm, the product from glycerin and TMDP) over time (Fig. S29†). Similarly, there are several decreasing peaks between ∼3.8 ppm and ∼4.3 ppm in the spectrum of ^1^H NMR (Fig. S30†), which confirms the results of ^31^P NMR.

### 3.3 Comparison of decomposition of different model compounds

**Fig. 7:**
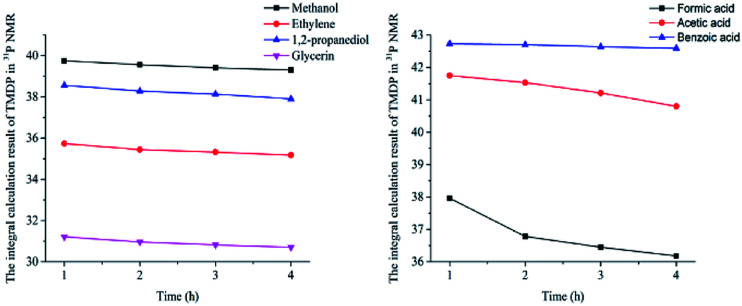
The integral calculation results of TMDP for different model compounds in ^31^P NMR (Left: sample added alcohols, Right: sample added carboxylic acids).

**Fig. 8:**
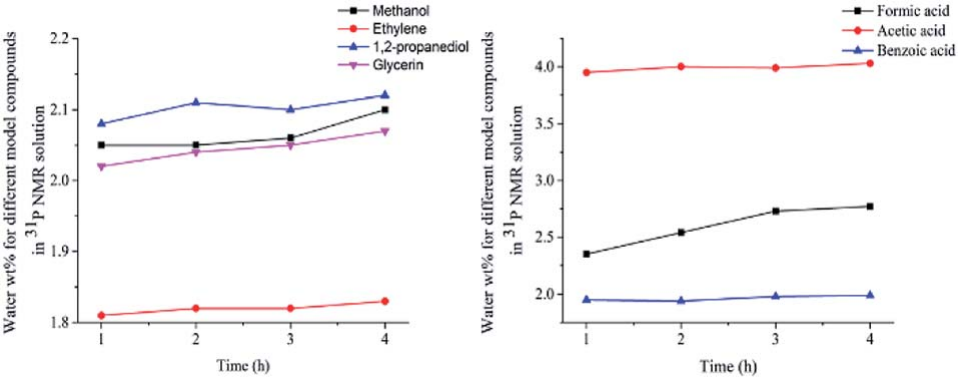
The content of water for different model compounds in ^31^P NMR solution (Left: sample added alcohols, Right: sample added carboxylic acids).

**Fig. 9:**
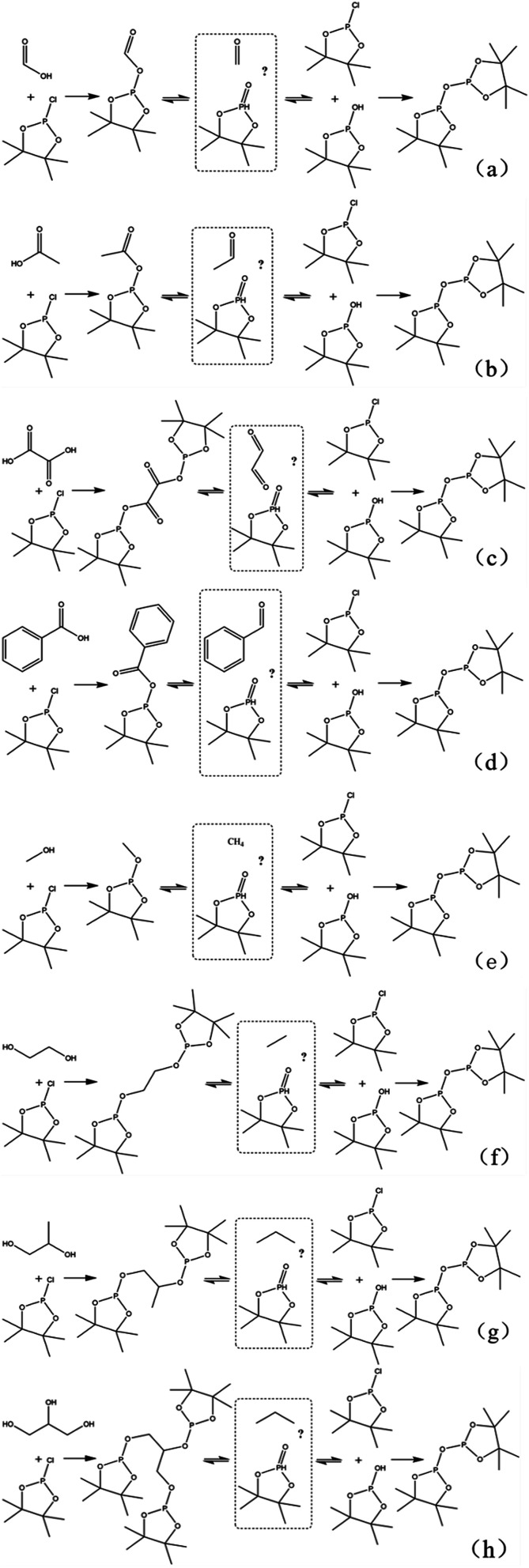
The pathway of decomposition of eight model compounds into water: (a) formic acid, (b) acetic acid, (c) oxalic acid, (d) benzoic acid, (e) methanol, (f) ethylene glycol, (g) 1,2-propanediol, (h) glycerin.

**Fig. 10:**
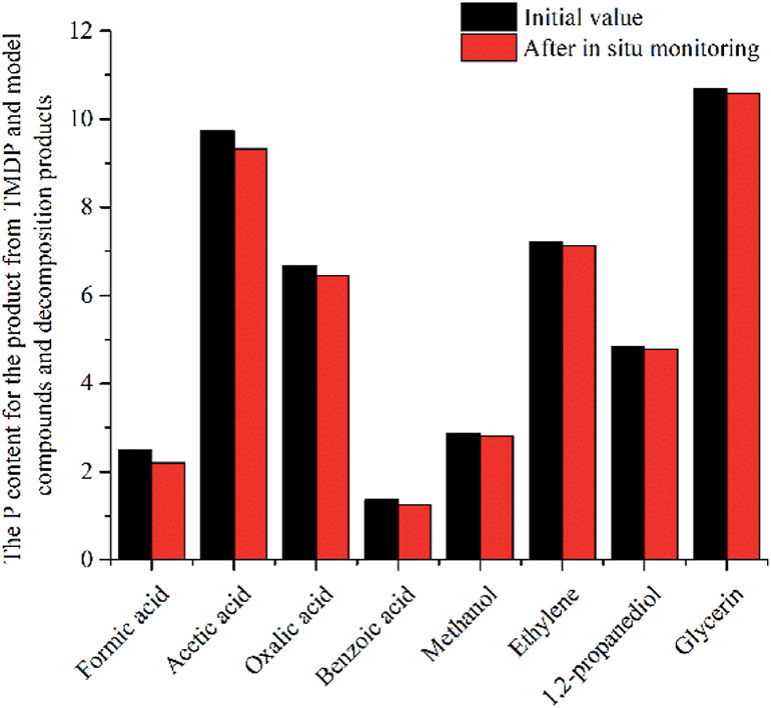
The P content for the product from TMDP and model compounds and decomposition products after *in situ* monitoring process.

In this study, accurate and quantitative characterization of four different alcohols and acid model compounds was completed. The results of changes in the content of these model compounds are shown in Table 3. The results show that regardless of the model compound, it decreased during the monitoring period. Since no precipitate formation was observed in the experiment, the decline trend was due to the decomposition of the compounds. As can be seen from the table, carboxylic acid compounds has poor stability in ^31^P NMR solution compared with the alcohol compounds. Each acid model compound was reduced by at least 10% after long-term storage, especially with almost complete decomposition of formic acid during the monitoring period. Furthermore, it is interesting to find that oxalic acid was extremely unstable and completely decomposed within one hour. Therefore, dibasic acids were more unstable than monobasic acids and can be decomposed rapidly. A possible cause is the presence of two carboxyl function groups. In addition, aromatic acid was relatively stable compared to aliphatic acids during the *ex situ* monitoring process. Acetic acid decreased by 39.55% after 14 days, while benzoic acid decreased by 17.37%. The presence of benzene ring increases the stability of aromatic acids. Several alcohols compounds were relatively stable and decomposed slowly, with a reduction of less than 10% shown in Table 3. The integral calculation results of TMDP for different samples in ^31^P NMR is shown in Fig. 7, which indicates that phosphorylation reagent was continue to consumed during the *in situ* monitoring period. The reason for this change is that new formed compounds from decomposition of the products from model compounds and TMDP can also react with TMDP. Furthermore, the results in Fig. 8 show an in increase in water content in all samples during the monitoring process, which indicates that water accounts for a large proportion of the model compounds decomposition products. These chemicals used in this study can be decomposed into water through the same reaction pathway, which has been proposed in the above analysis (Fig. 9). Moreover, in addition to water formation, these model compounds decompose to form the corresponding aldehyde compounds (Fig. 9). In addition, it was found in Fig. 10 that the amount of the initial P-containing product from TMDP and model compounds was more than the sum of the decomposition product and the derived compounds after short-term storage. The reason for this change is the formation of gases or compounds without hydroxyl groups, such as formaldehyde (Fig. 9).

Degree of decline in eight model compounds during 4 hours of *in situ* and 14 days of *ex situ* stability tests with TPPO as internal standard

**Table:** 

Time	Formic acid^a^	Acetic acid^a^	Oxalic acid^a^	Benzoic acid^a^
1 h	5.78%	0.97%	100%	1.48%
2 h	11.98%	1.79%	—	3.06%
3 h	20.13%	2.60%	—	4.65%
4 h	29.43%	3.21%	—	6.24%
1 d	95.51%	10.74%	—	8.63%
7 d	98.45%	27.54%	—	10.22%
14 d	99.22%	39.55%	—	17.37%

a The percentage calculation method: (the amount of model compounds added to the initial sample − the amount of model compounds detected)/the amount of model compounds added to the initial sample.

**Table:** 

Time	Methanol^a^	Ethylene glycol^a^	1,2-propanediol^a^	Glycerin^a^
1 h	0.64%	0.28%	0.57%	0.70%
2 h	0.99%	0.56%	1.19%	1.63%
3 h	1.33%	0.97%	1.60%	2.10%
4 h	1.68%	1.25%	2.01%	2.56%
1 d	3.77%	1.81%	2.42%	3.86%
7 d	5.85%	3.88%	4.67%	4.51%
14 d	6.20%	4.44%	5.90%	4.89%

Surprisingly, in addition to the peaks of water, the peaks at ∼0.19 ppm and ∼3.74 ppm were found to form and gradually increase over a long period of time in the results of *ex situ*^31^P NMR monitoring of all model compounds other than oxalic acid. A sample without any model compound added was test by quantitative ^31^P NMR before and after 7 days storage. The spectral result is shown in Fig. S31,† which shows that two peaks located at ∼0.19 ppm and ∼3.74 ppm formed after long-term storage. It indicates the compounds represented by these peaks were not formed by decomposition of the model compounds used in this study ^31^P NMR solution, which is caused by the following two possible reasons. One possible reason is that the main impurities in the solution gradually formed a compound with hydroxyl group after a long period of time and underwent a derivatization reaction with TMDP, which is difficult to determine. Another reason may be due to some small amounts of chemicals in the ^31^P NMR solution decomposing after long-term storage, but this is not caused by TPPO with its stability proven in previous study.^32^ Although the compounds specifically represented by the peaks at ∼0.19 ppm and ∼3.74 ppm in the ^31^P NMR were difficult to determine, it was confirmed that this phenomenon was due to the original solution. As can be seen from Table 1, 2 and S6–S11,† in addition to the integral regions in which the model compounds is located, the region of phenolic hydroxyl group has a small integral value, which is extremely small relative to other regions of interest. The content of phenolic hydroxyl groups is a few hundredths or even a few thousandths of the amount of the model compounds used, and the changes were much less dramatic. Moreover, the content had an irregular change rule, which shows that its content would increase and decrease as time increases. In addition, there was no peak formed within the integral region of phenolic hydroxyl group in the ^31^P NMR of all model compounds studied. A sample without any model compound added was test by *in situ*^31^P NMR monitoring. The spectral result is shown in Fig. S32† and the integral result of phenolic hydroxyl group is shown in Table S12.† Interestingly, even in the absence of the model compounds, the ^31^P NMR solution also had the presence of phenolic hydroxyl group, which is reflected in the results of Table S12.† Similarly, there was no peak formed in the region of phenolic OH in the upper left corner in Fig. S32.†*In situ*^31^P NMR monitoring results of all model compounds also show no peak in the phenolic hydroxyl region. In addition, it can be seen from *in situ*^1^H NMR monitoring result (Fig. S33†) that there was no new peak formation except for the original peak. This change in phenolic hydroxyl groups may be due to trace impurities in the solution, which is too small. These trace impurities may decompose by themselves or may react with some compounds in the solution. Moreover, the changes are also different due to the difference in the added model compounds. However, this is rare compared to the model compound itself. Therefore, this phenomenon is caused by many reasons, which makes it difficult to determine what compound it is.

In summary, the optimum test and preparation time of the sample can be determined by accurate and quantitative ^31^P NMR characterization. The high oxygen content of pyrolysis oil hinders its application. Therefore, quantitative and accurate characterization of different oxygen-containing functional groups is of great importance. To further investigate the properties of pyrolysis oils, studies on the stability of different model compounds representing pyrolysis oils are promising and necessary.

## 4. Conclusions

In this study, an in-depth investigation has been completed by using ^31^P NMR, which is a method that can achieve the quantitative characterization of different hydroxyl functional groups. Several model compounds containing hydroxyl functional groups including alcohols and carboxylic acids have been studied by both short time (4 hours) *in situ* and long-time (1 day, 7 days and 14 days) *ex situ* monitoring. These results obtained indicate that carboxylic acids compounds are unstable and gradually decompose during the monitoring period, as well as alcohols. However, all the carboxylic acids have poorer stability than all alcohols. For carboxylic acids, aromatic acids are relatively more stable than aliphatic acids. Interestingly, oxalic acid is extremely unstable and completely decomposed in the first hour, while formic acid has only a small amount left in a day. The alcohol model compounds used in this study maintain a content of more than 90% after long-term storage. In addition, the water content is also unstable during the real-time monitoring process. Moreover, water is found in experiments as the main product of decomposition of these model compounds. Depending on the time of stabilization of the various compounds shown in the results, the optimal time for testing and preparation needs to be considered to achieve the accuracy and quantification of the study. This is a report on the quantitative characterization of different oxygen-containing functional groups representing pyrolytic oils and the first study on the similarities and differences of the decomposition of carboxylic acids and alcohols in ^31^P NMR solutions. The results of this in-depth investigation can provide important assistance in the study of the upgrading of pyrolysis oil and its application.

## Conflicts of interest

The authors declare that the research was conducted in the absence of any commercial or financial relationships that could be construed as a potential conflict of interest.

## Supplementary Material

RA-009-C9RA04099D-s001
