# Supplementary material for: In-depth study on the effect of oxygen-containing functional groups in pyrolysis oil by P-31 NMR
Source: RSC Adv. 2019 Aug 29;9(47):27157–66. doi: 10.1039/c9ra04099d (PMC9070637; doi:10.1039/c9ra04099d)
Supplement: RA-009-C9RA04099D-s001 [file RA-009-C9RA04099D-s001.pdf]

Electronic Supplementary Material (ESI) for RSC Advances.
This journal is © The Royal Society of Chemistry 2019

Supporting information

In-depth study on the effect of oxygen-containing functional groups in pyrolysis oil by P-31 NMR

Zhihong Wu[1,2], Haoxi Ben[1,2,*], Yunyi Yang[1,2], Ying Luo[1,2], Kai Nie[3,4], Wei Jiang[3,4], Guangting Han[3,4]

[1] Southeast University, Nanjing 210096, China

[2] Key Laboratory of Energy Thermal Conversion and Control of Ministry of Education, School of Energy and Environment, Nanjing 210096, China

[3] Qingdao University, Qingdao 266071, China

[4] State Key Laboratory of Bio-Fibers and Eco-Textiles, Qingdao University, Qingdao 266071, China

*Correspondence should be addressed to Pro. Haoxi Ben

Email: benhaoxi@gmail.com

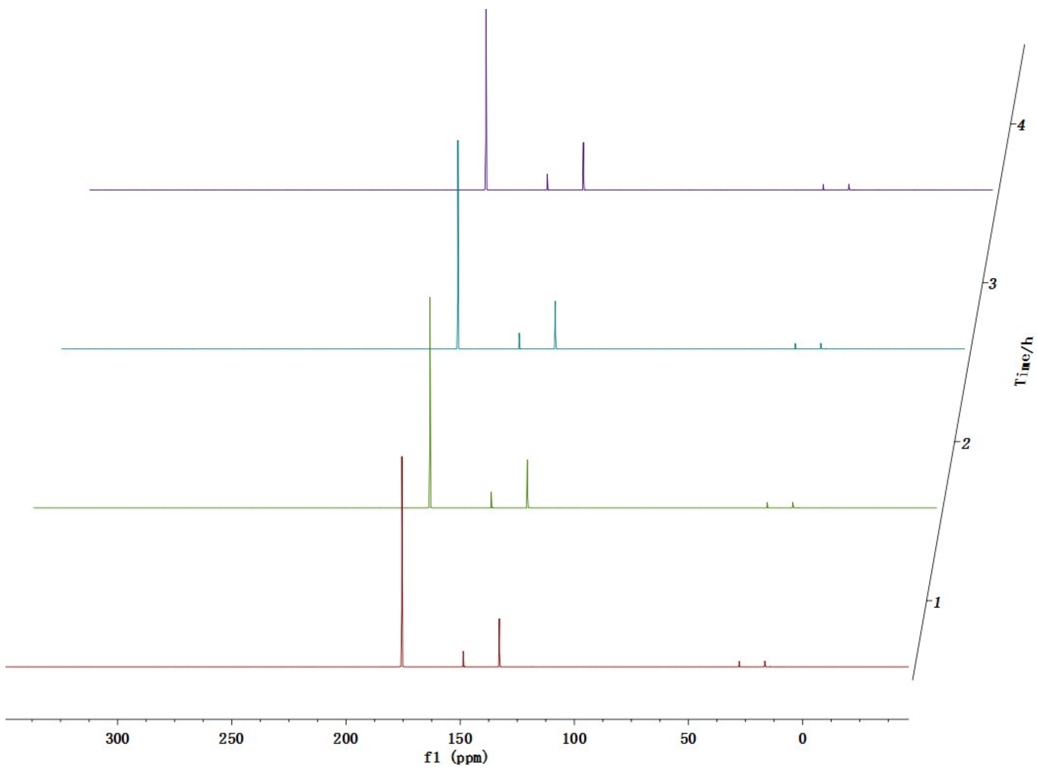

a

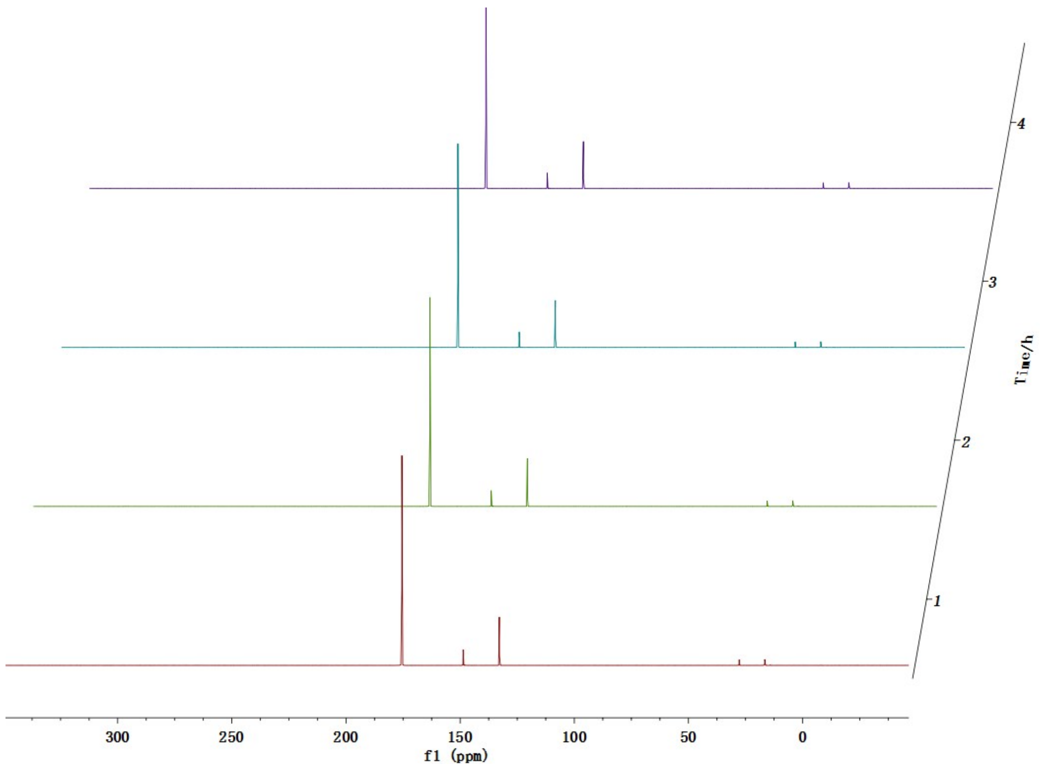

b

Fig. S1 In situ monitoring for the stability of formic acid with different D1 in ^{31}P NMR (a: D1=25s; b: D1=100s).

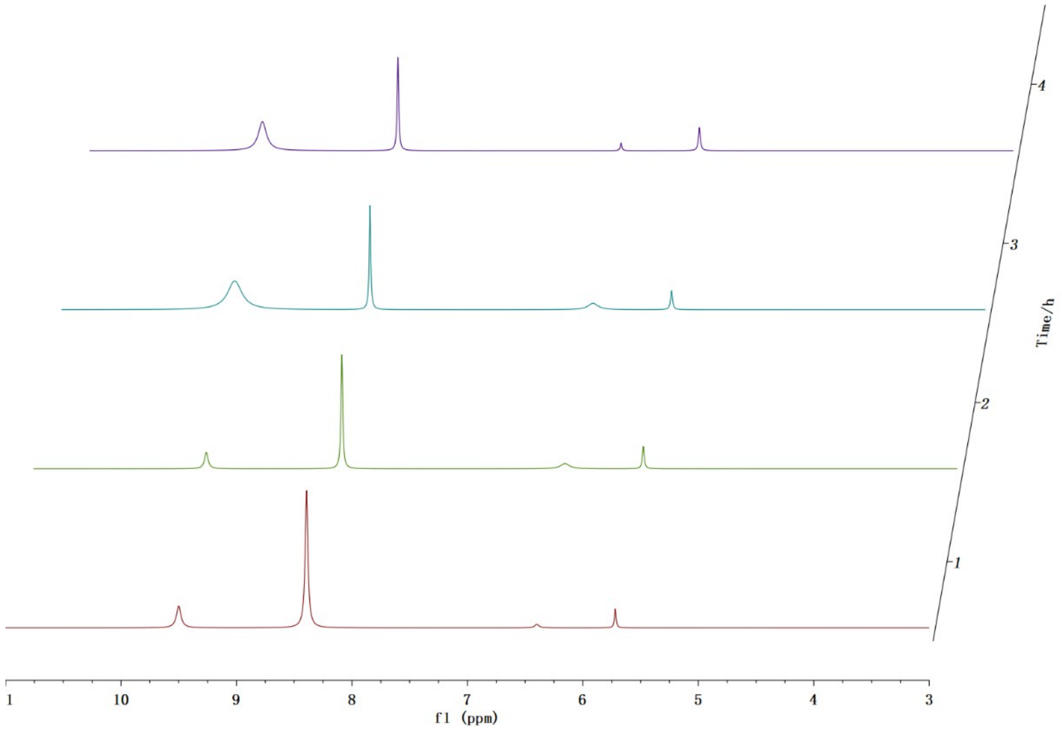

Fig. S2 In situ monitoring for the stability of formic acid in ^{1}H NMR.

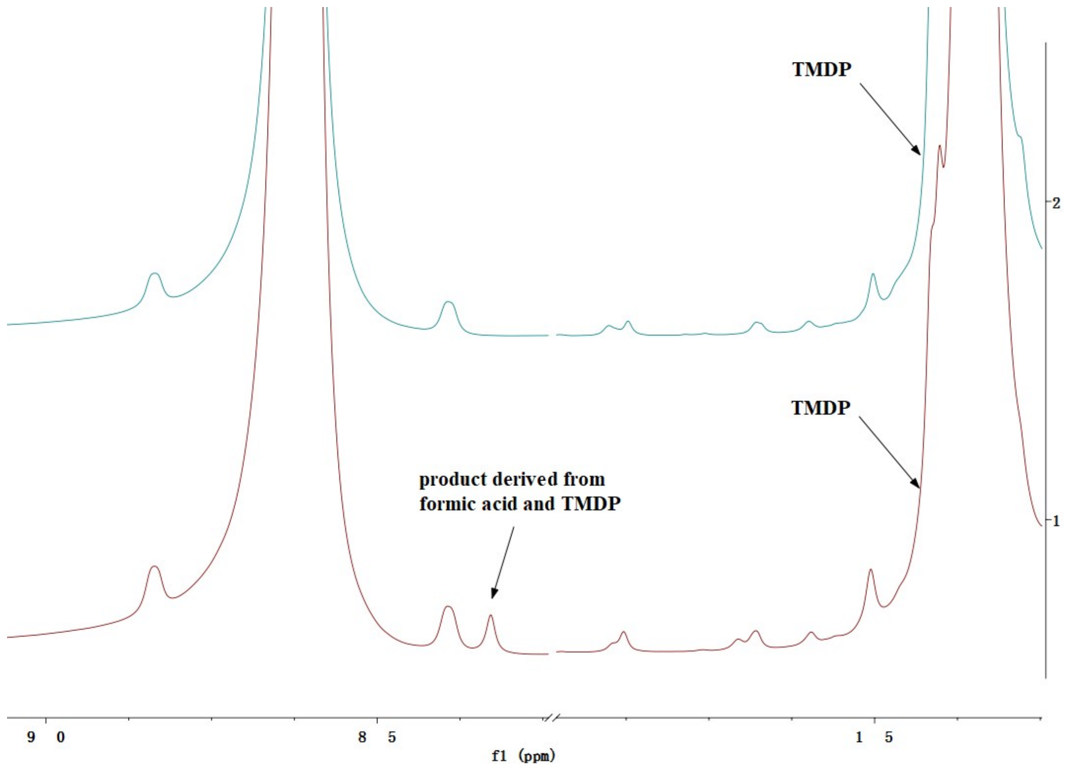

Fig. S3 Comparison before(bottom) and after(top) 14 days of ex situ monitoring of formic acid in ^{1}H NMR.

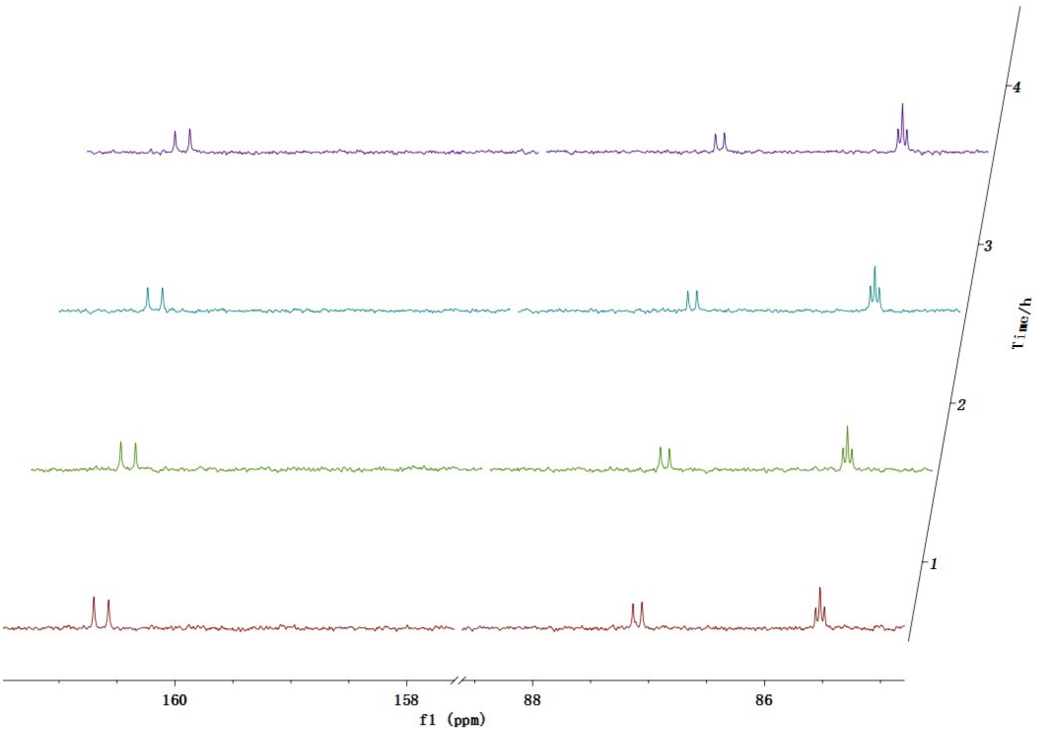

F

ig. S4 The peak of formic acid and some growing peaks during the in situ monitoring in ^{13}C NMR with TPPO as internal standard.

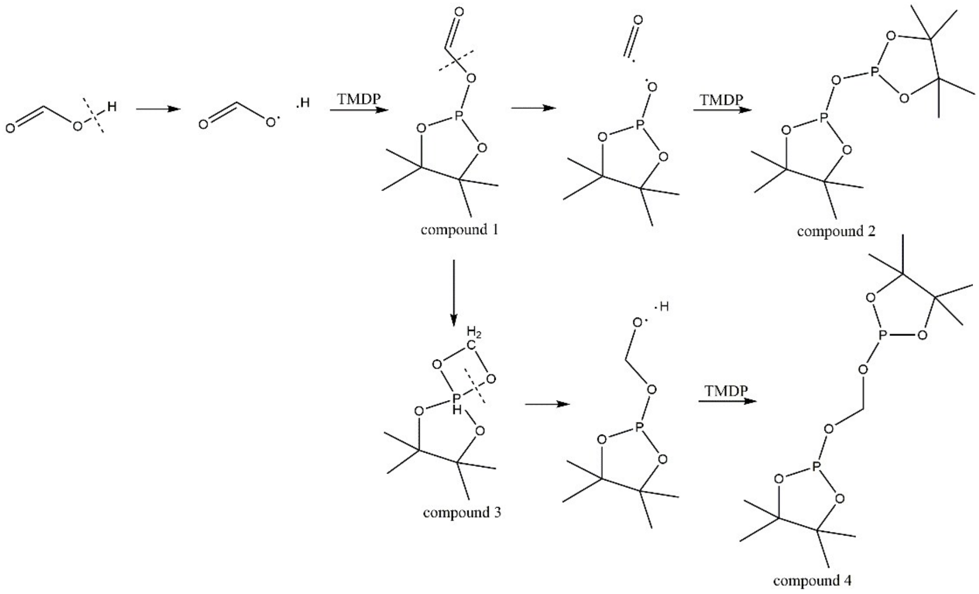

Fig S5. Two possible decomposition pathway for formic acid in ^{31}P NMR.

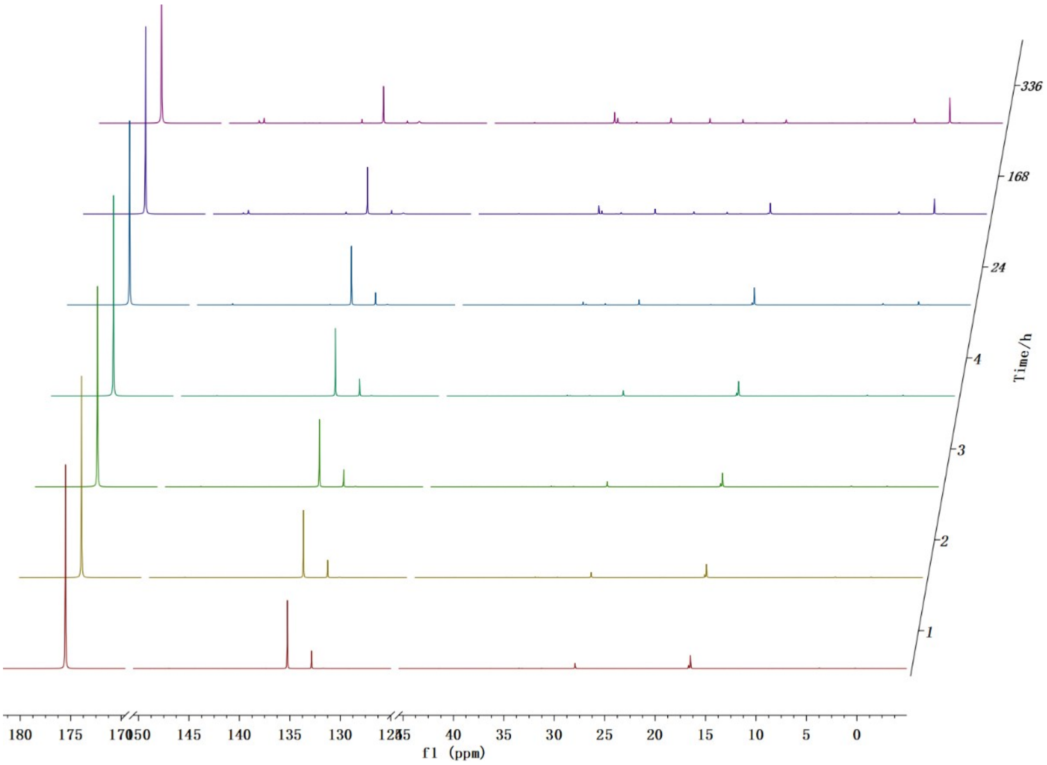

Fig. S6 In situ and ex situ monitoring for the stability of acetic acid during ^{31}P NMR with TPPO as internal standard.

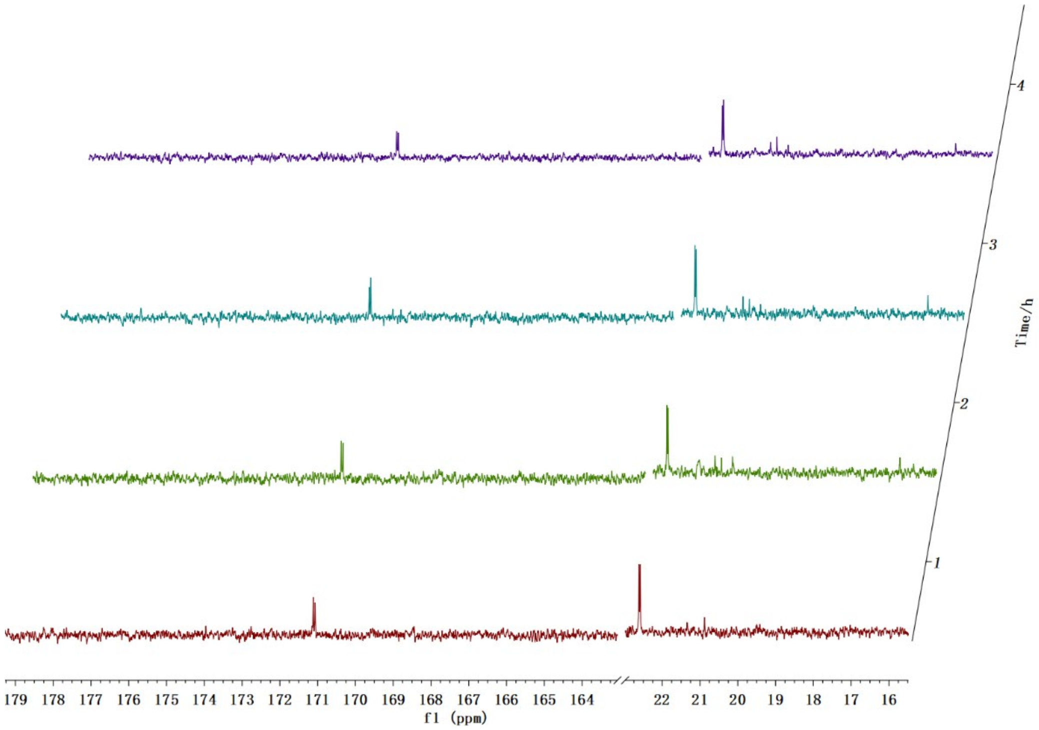

F

ig. S7 The peak of acetic acid during the in situ monitoring in ^{13}C NMR with TPPO as internal standard.

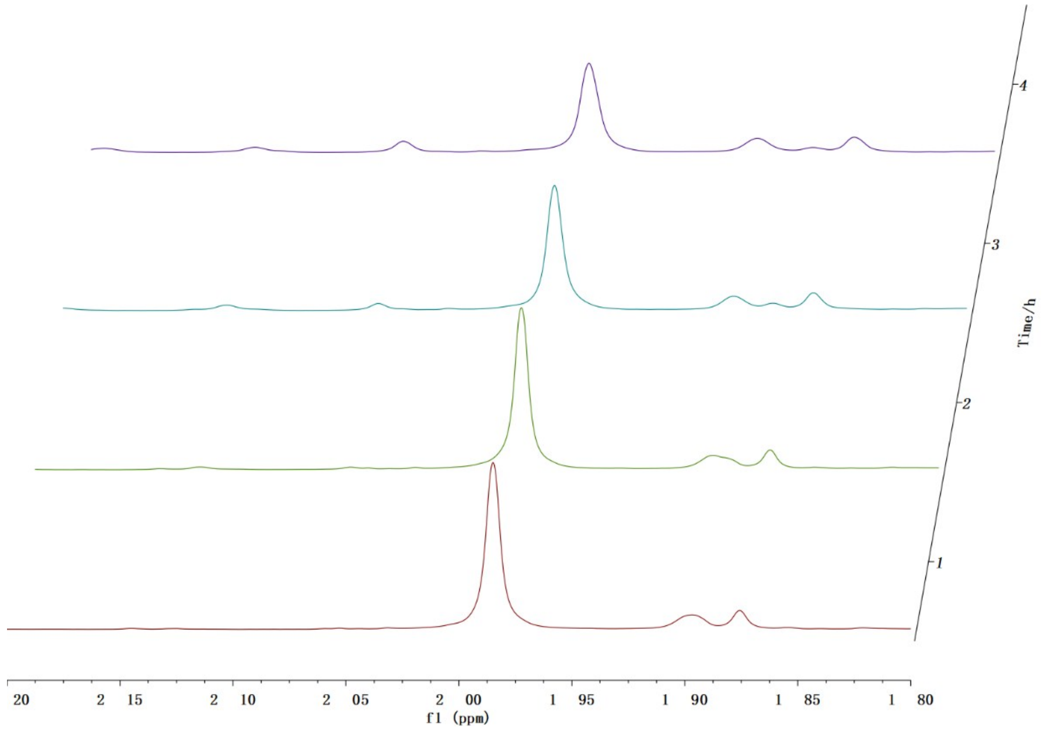

Fig. S8 In situ monitoring for the stability of acetic acid in ^{1}H NMR.

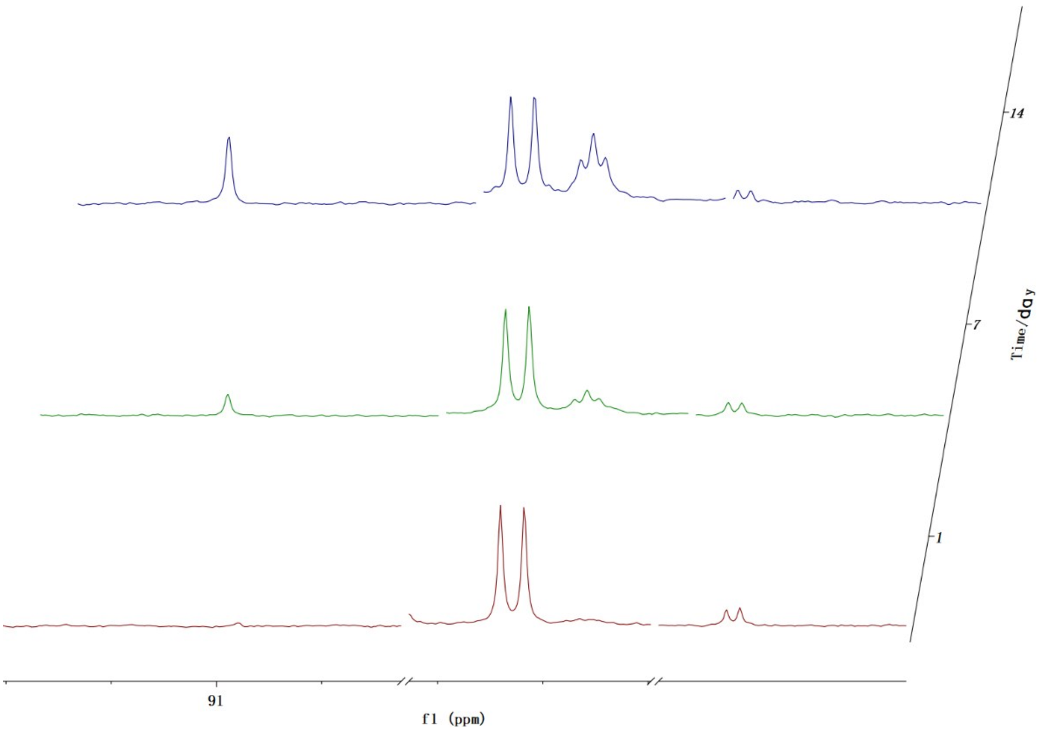

F

ig. S9 Ex situ monitoring for the stability of acetic acid in ^{13}C NMR.

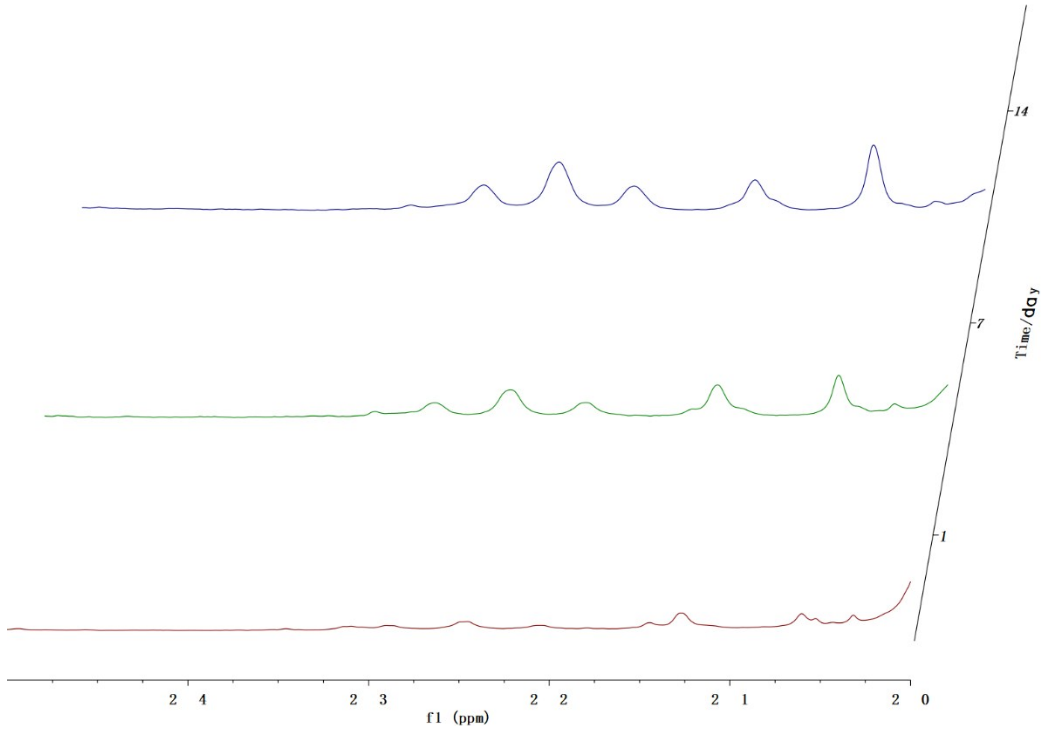

Fig. S10 Ex situ monitoring for the stability of acetic acid in ^{1}H NMR.

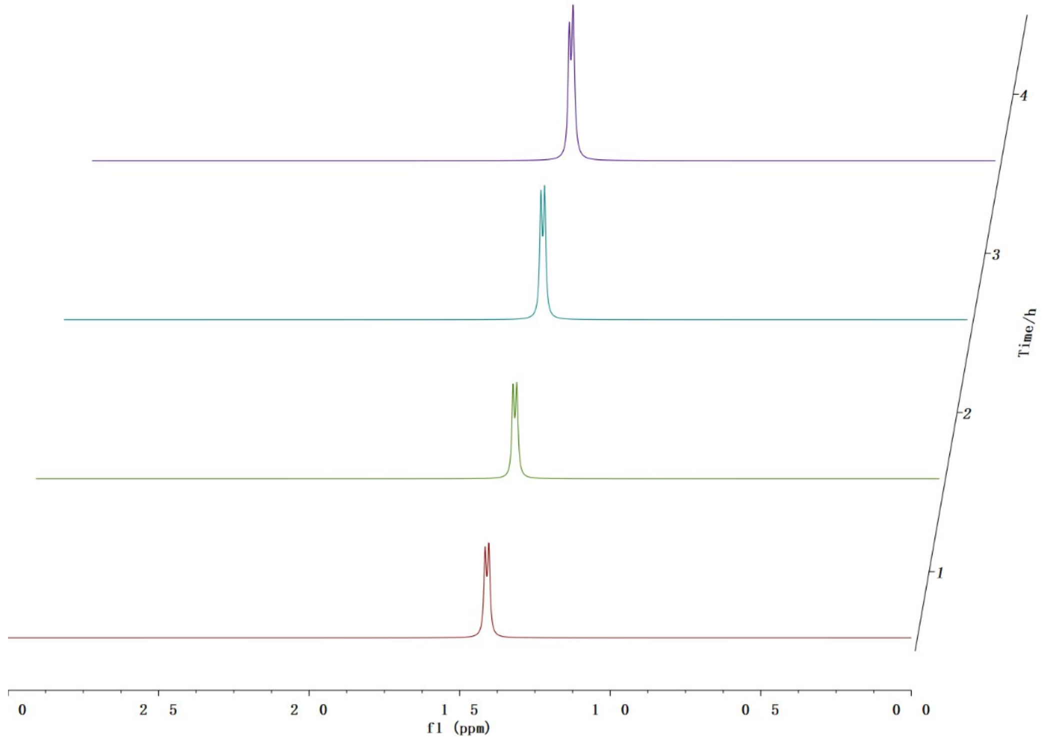

Fig. S11 The growing peak during the in situ monitoring of acetic acid in ^{1}H NMR with TPPO as internal standard.

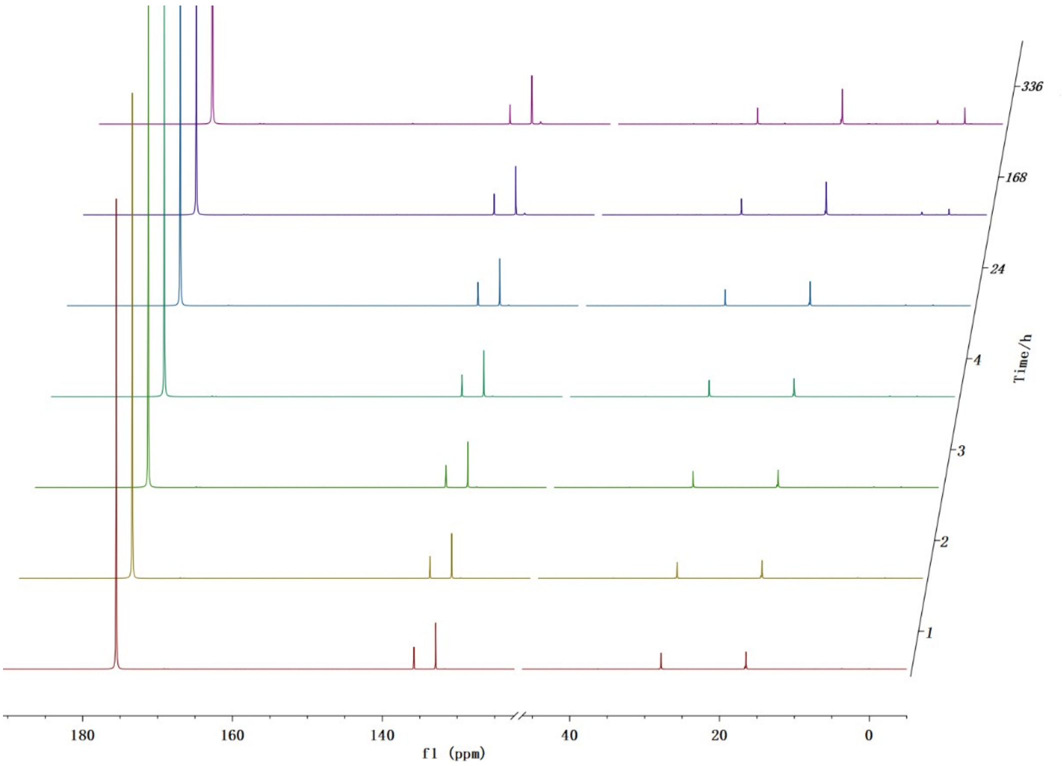

Fig. S12 In situ and ex situ monitoring for the stability of benzoic acid during ^{31}P NMR with TPPO as internal standard.

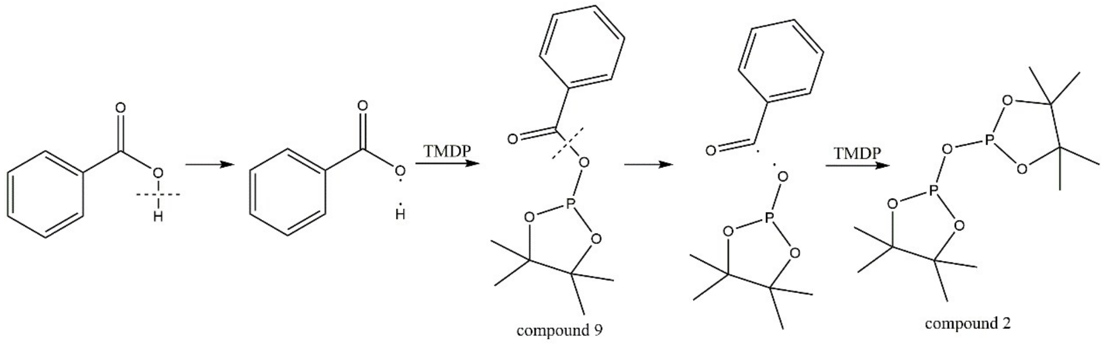

Fig. S13 One possible decomposition pathway for benzoic acid in ^{31}P NMR.

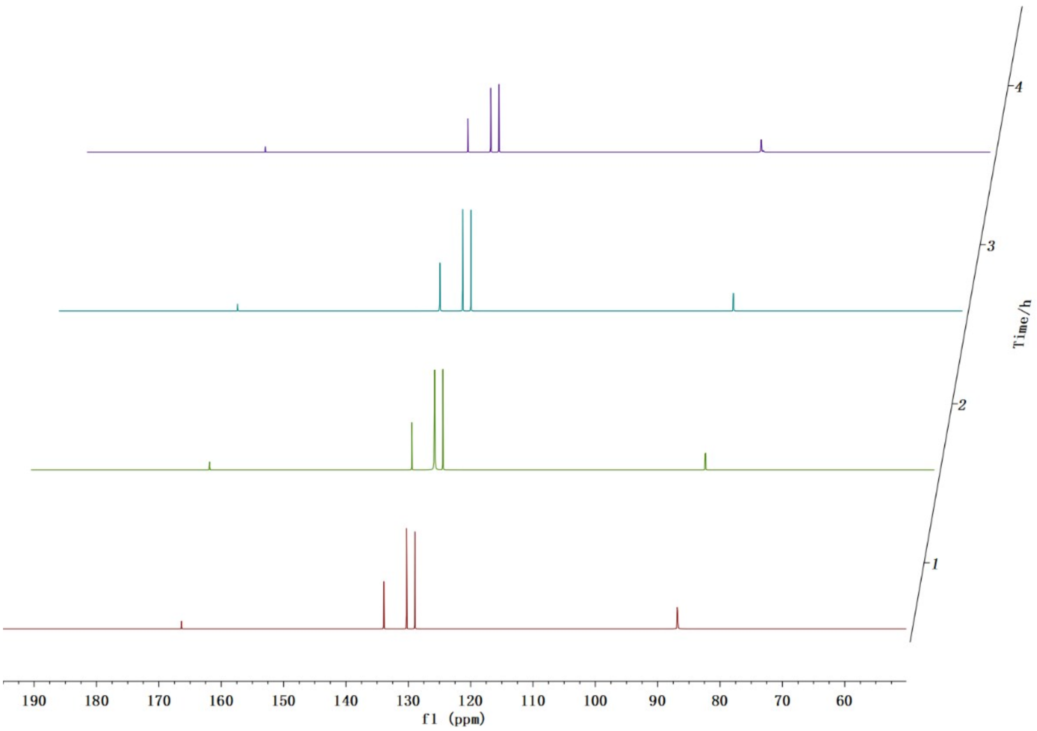

F

ig. S14 The peaks of benzoic acid during the in situ monitoring in ^{13}C NMR with TPPO as internal standard.

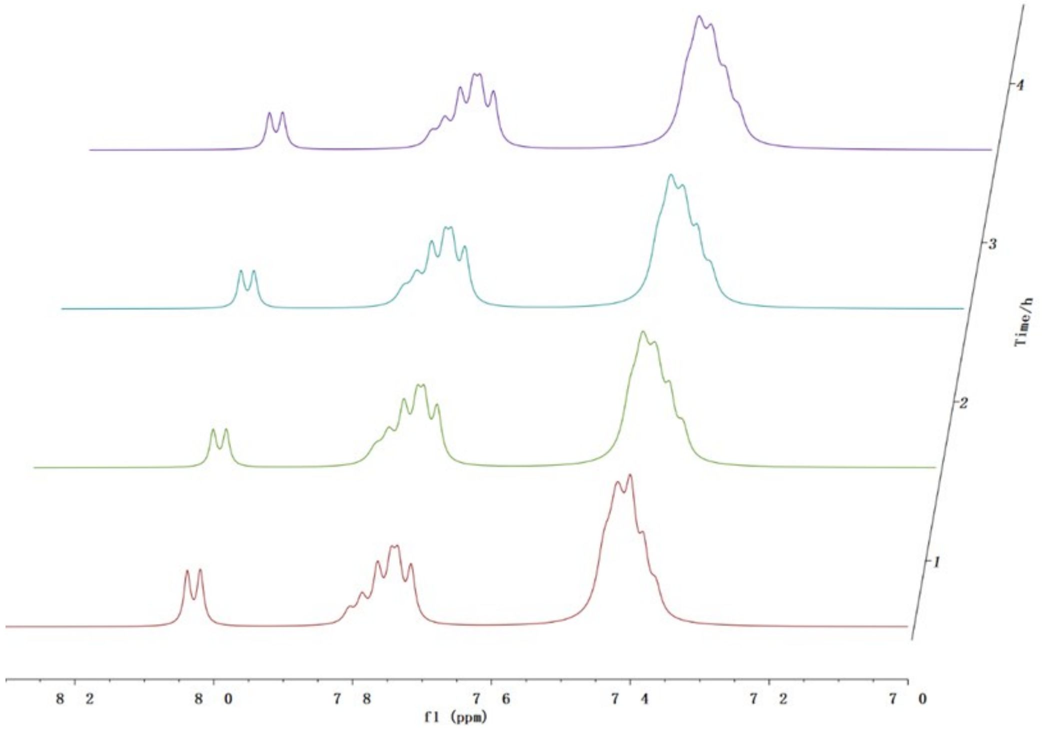

Fig. S15 The peaks of benzoic acid during the in situ monitoring in ^{1}H NMR with TPPO as internal standard.

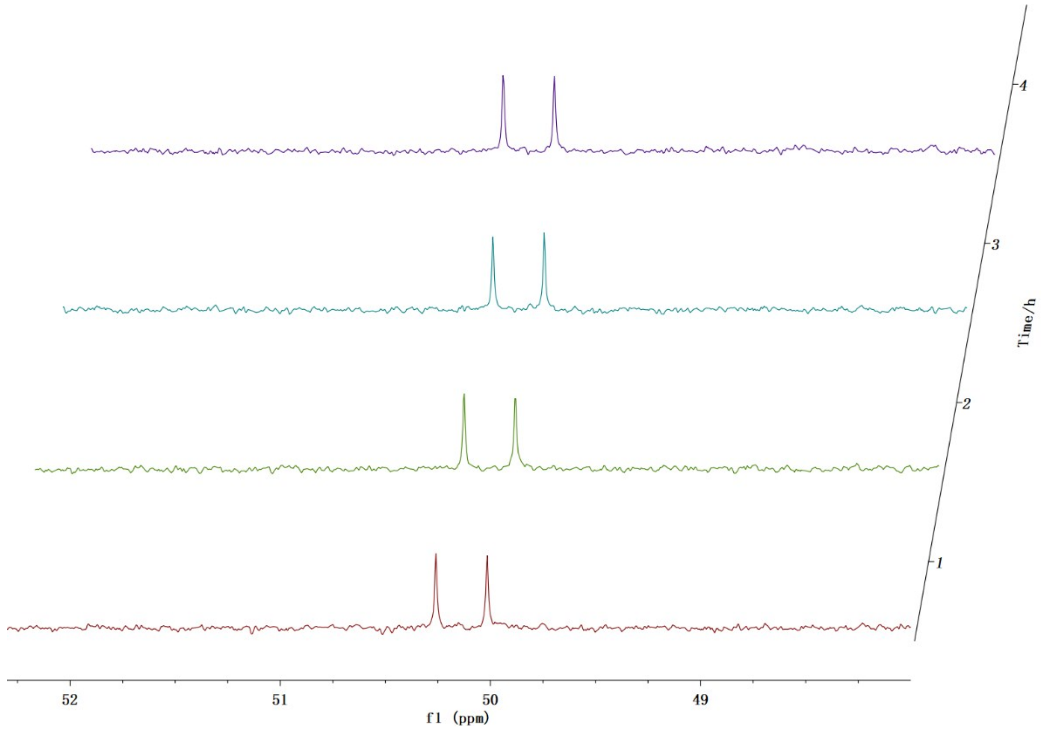

Fig. S16 The peak of methanol during the in situ monitoring in ^{13}C NMR with TPPO as internal standard.

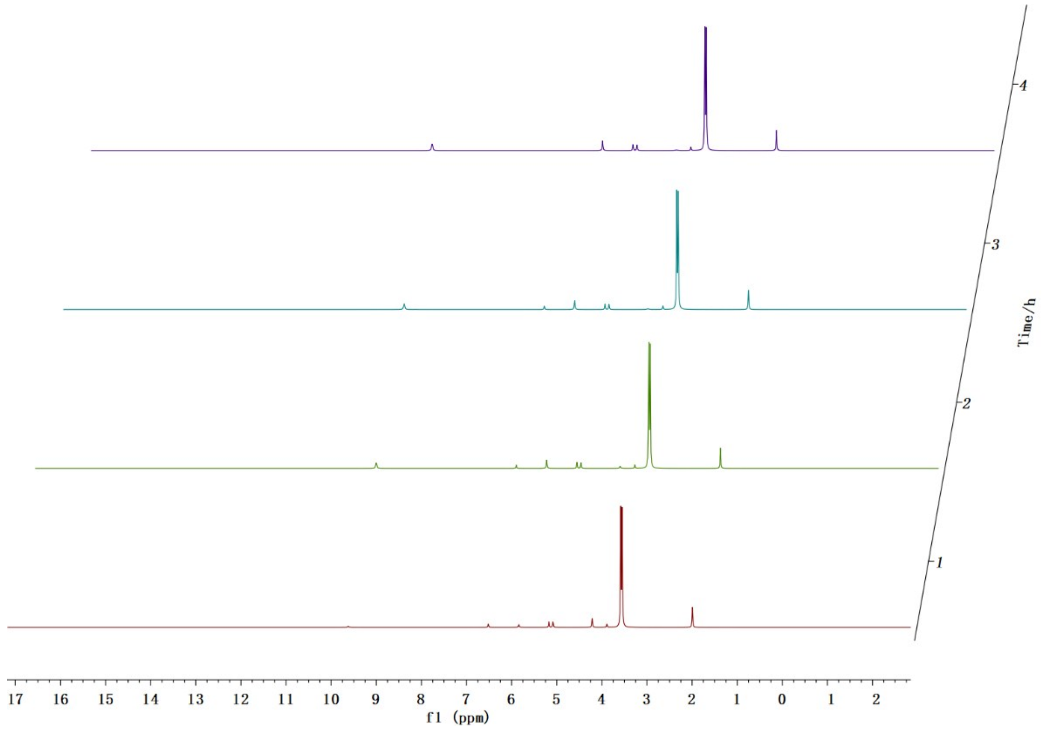

Fig. S17 The peak of methanol during the in situ monitoring in ^{1}H NMR with TPPO as internal standard.

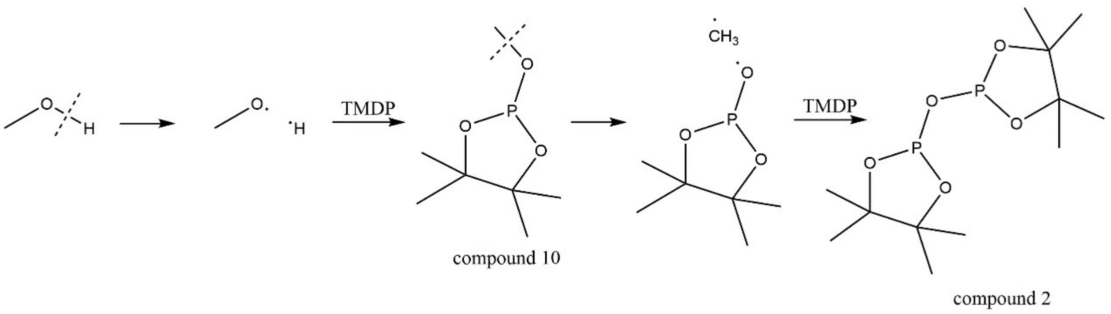

Fig S18. The major decomposition pathway for methanol in ^{31}P NMR.

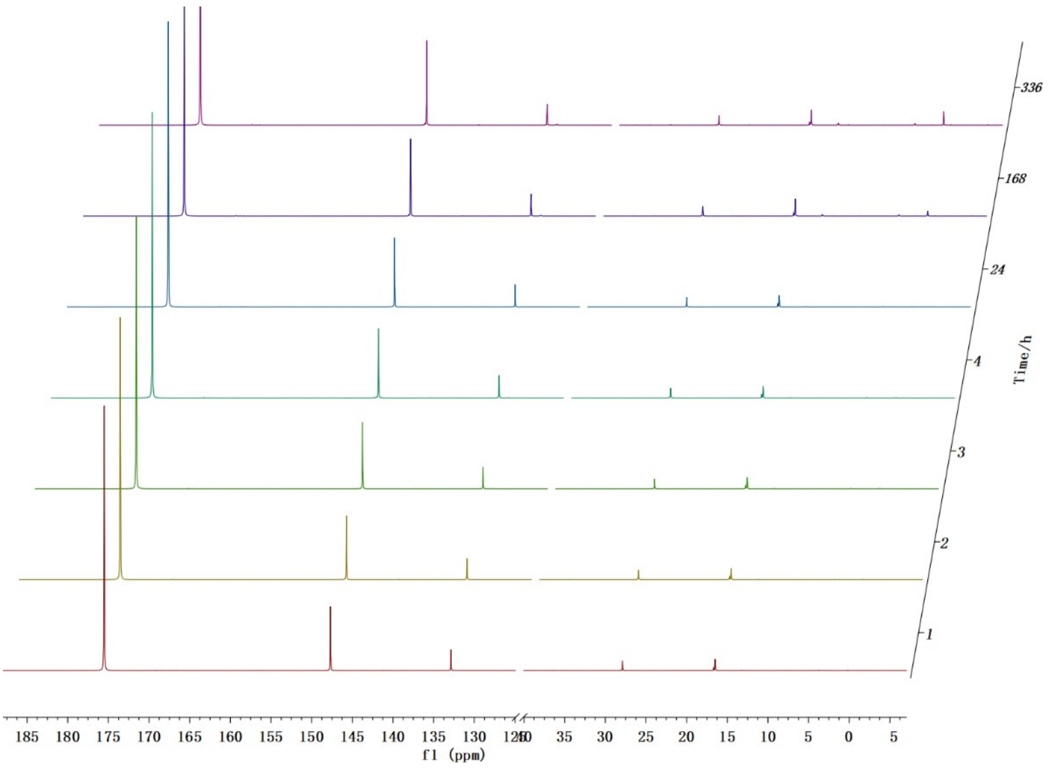

Fig. S19 In situ and ex situ monitoring for the stability of ethylene glycol during 31P NMR with TPPO as internal standard.

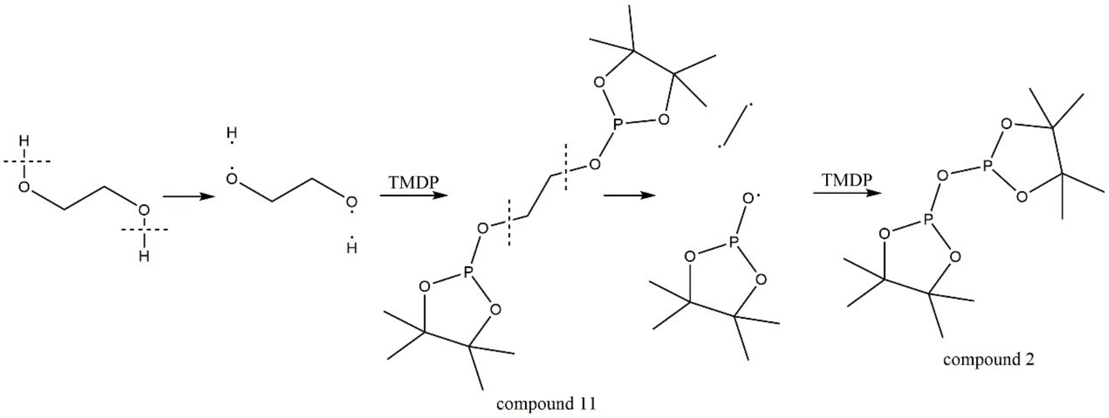

Fig. S20 The major decomposition pathway for ethylene glycol in ^{31}P NMR.

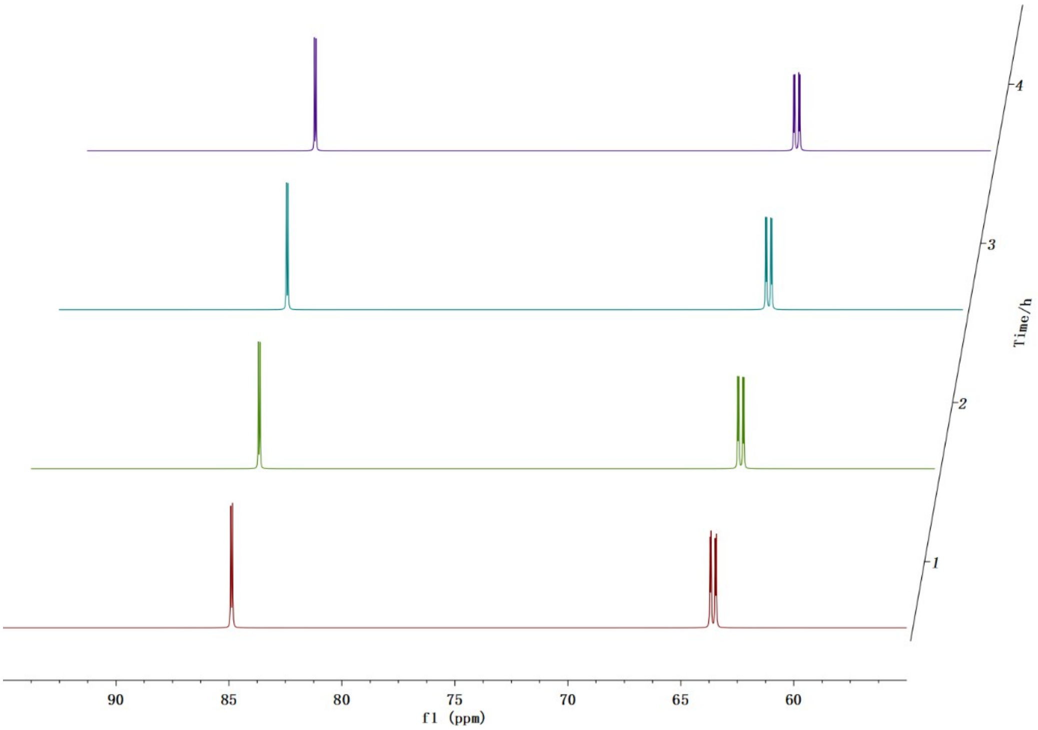

Fig. S21 The peaks of ethylene glycol during the in situ monitoring in ^{13}C NMR with TPPO as internal standard.

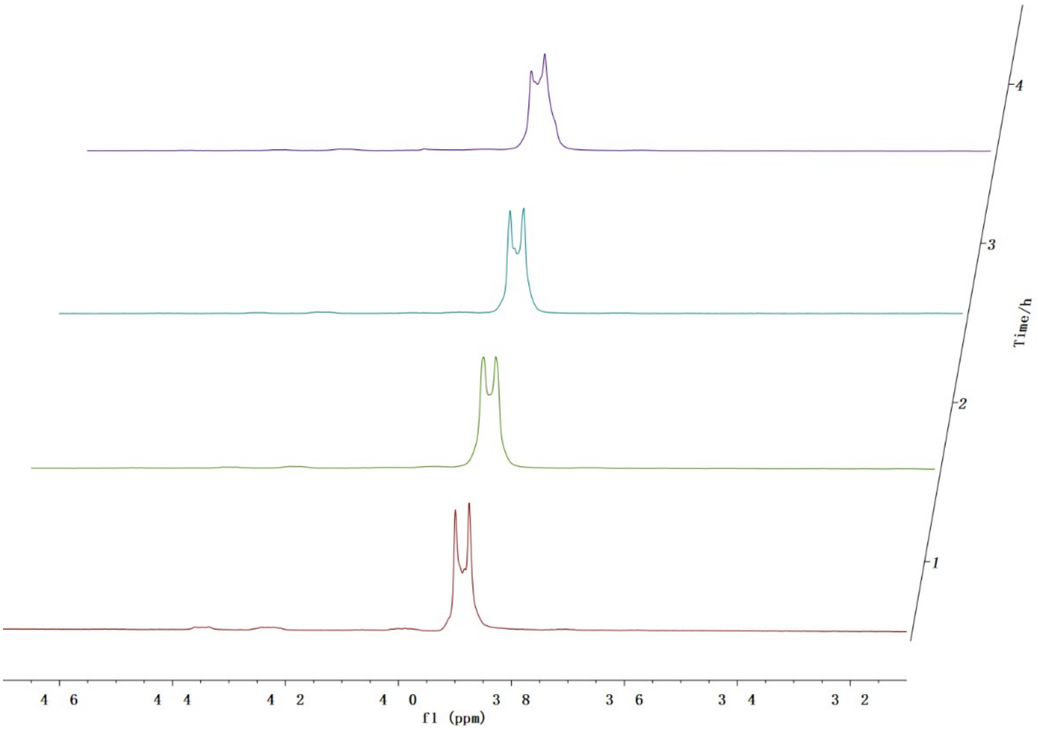

Fig. S22 The peak of ethylene glycol during the in situ monitoring in ^{1}H NMR with TPPO as internal standard.

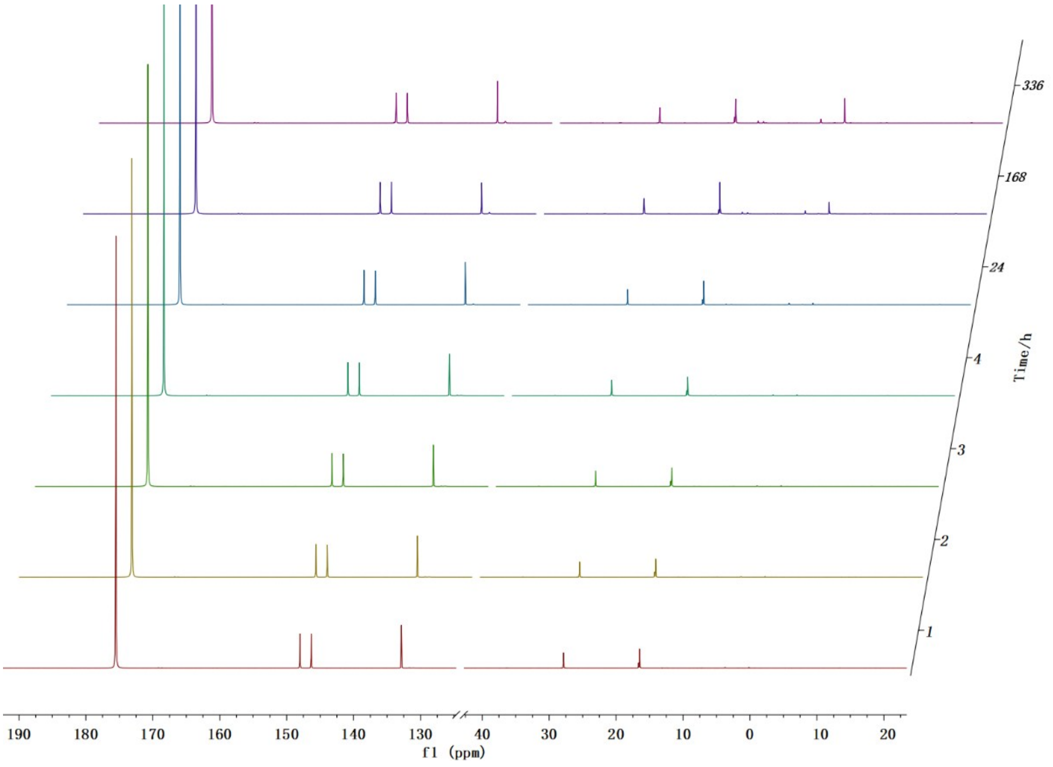

Fig. S23 In situ and ex situ monitoring for the stability of 1,2 propanediol during 31P NMR with TPPO as internal standard.

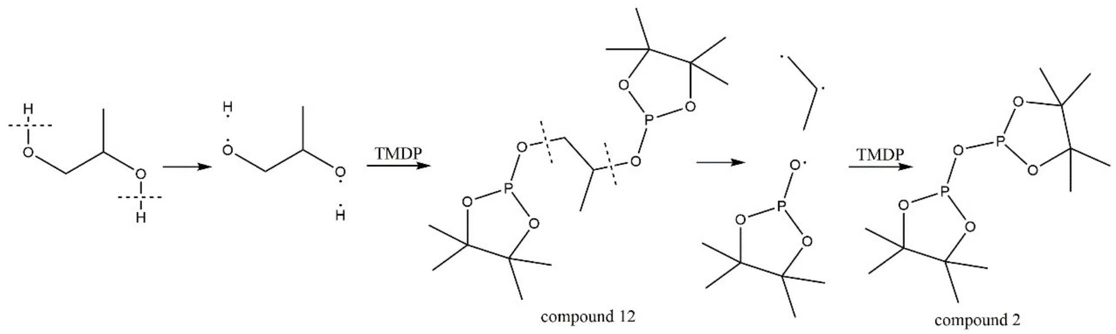

Fig. S24 The major decomposition pathway for 1,2-propanediol in ^{31}P NMR.

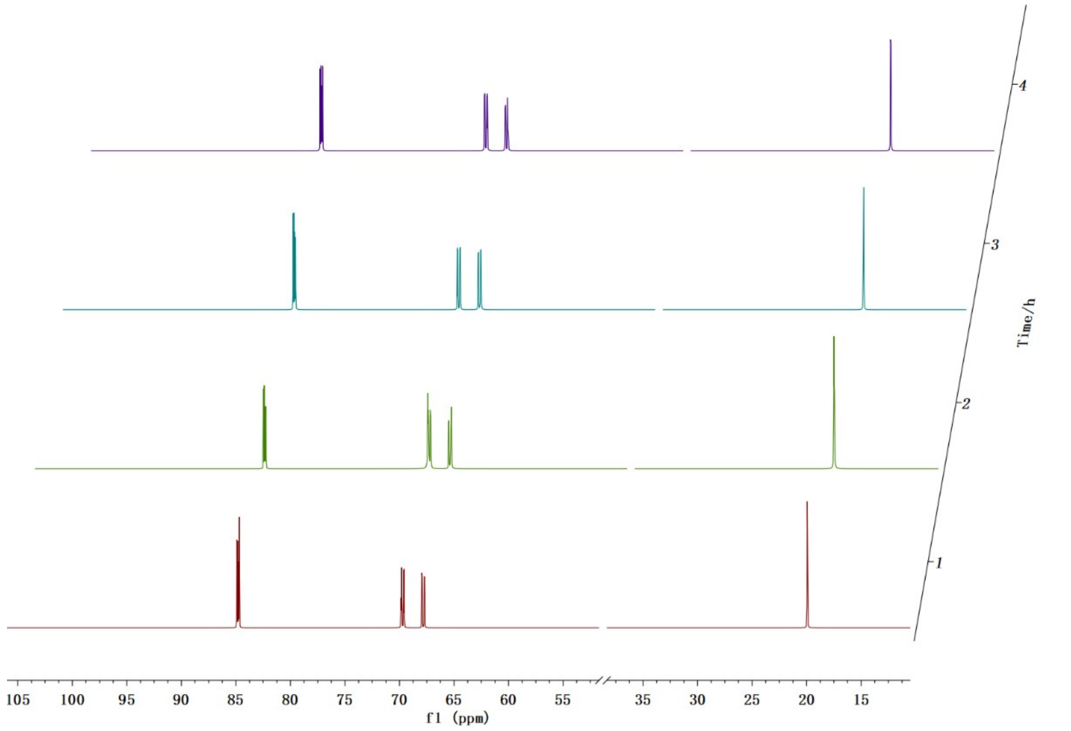

Fig. S25 The peaks of 1,2-propanediol during the in situ monitoring in ^{13}C NMR with TPPO as internal standard.

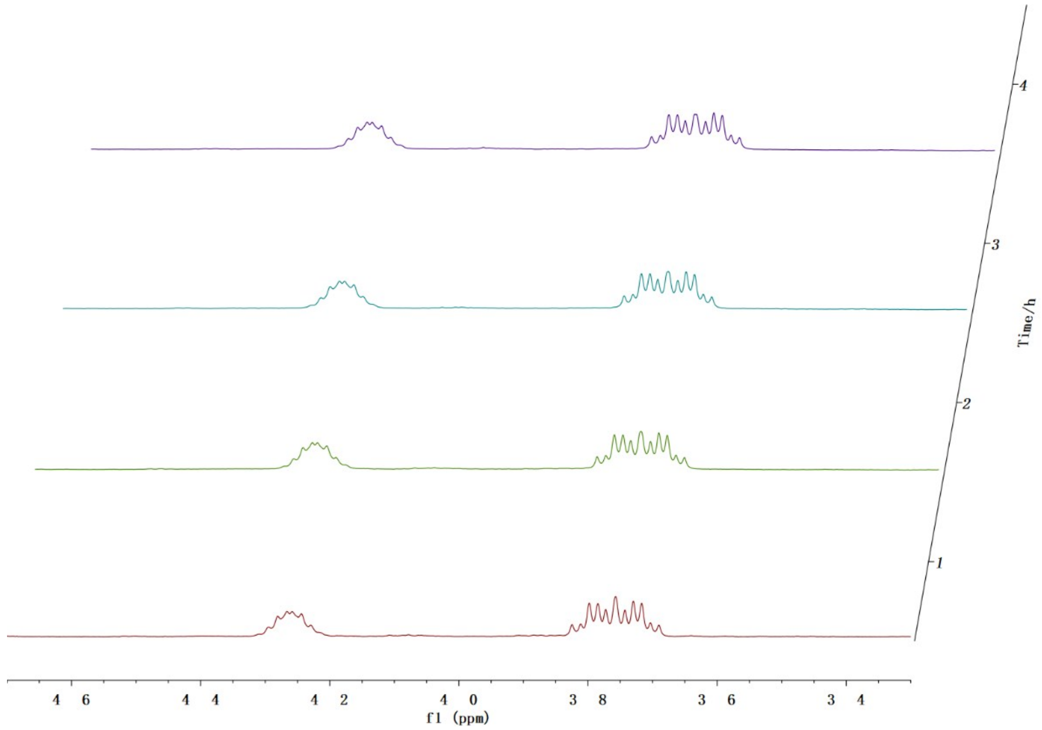

Fig. S26 The peaks of 1,2-propanediol during the in situ monitoring in ^{1}H NMR with TPPO as internal standard.

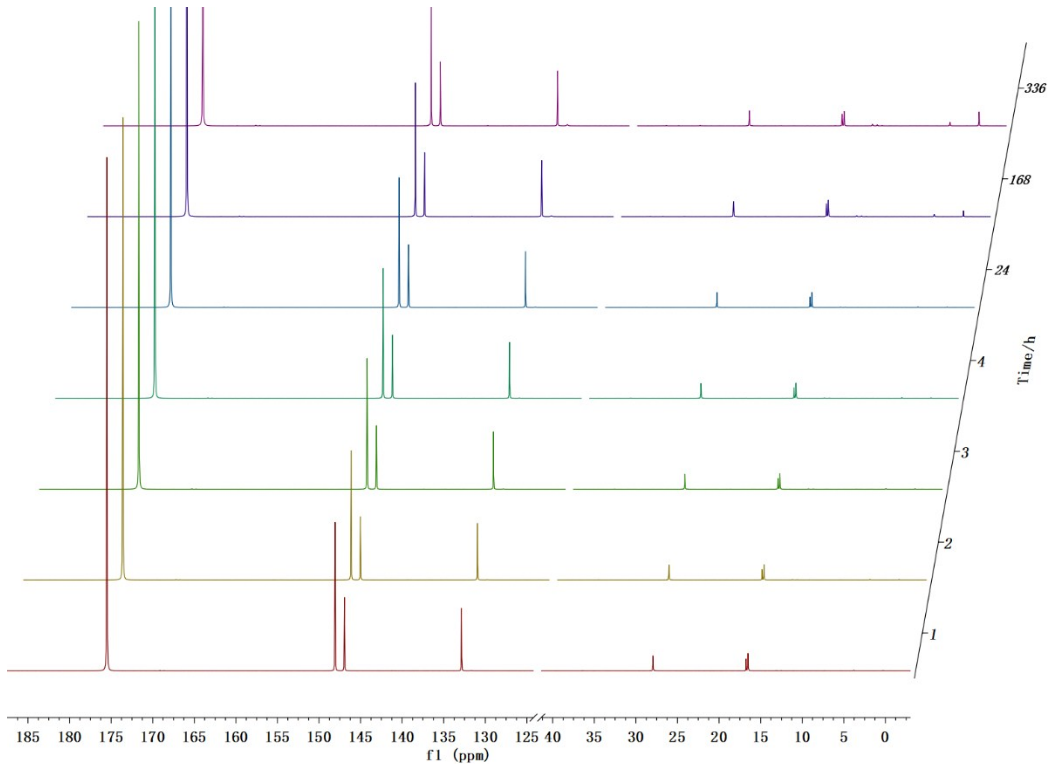

Fig. S27 In situ and ex situ monitoring for the stability of glycerin during ^{31}P NMR with TPPO as internal standard.

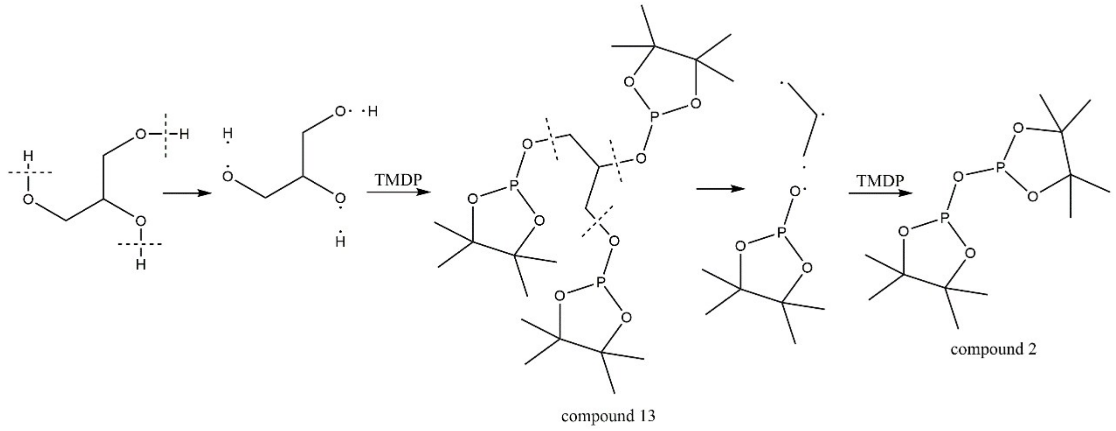

Fig. S28 The major decomposition pathway for glycerin in ^{31}P NMR.

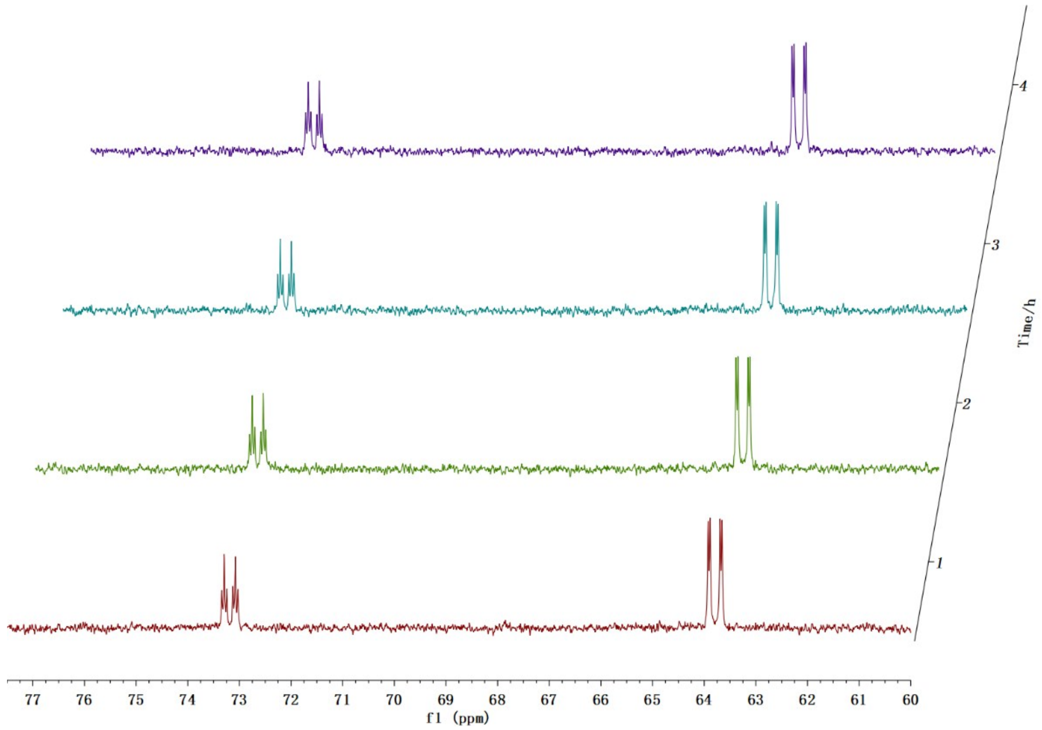

Fig. S29 The peaks of glycerin during the in situ monitoring in ^{13}C NMR with TPPO as internal standard.

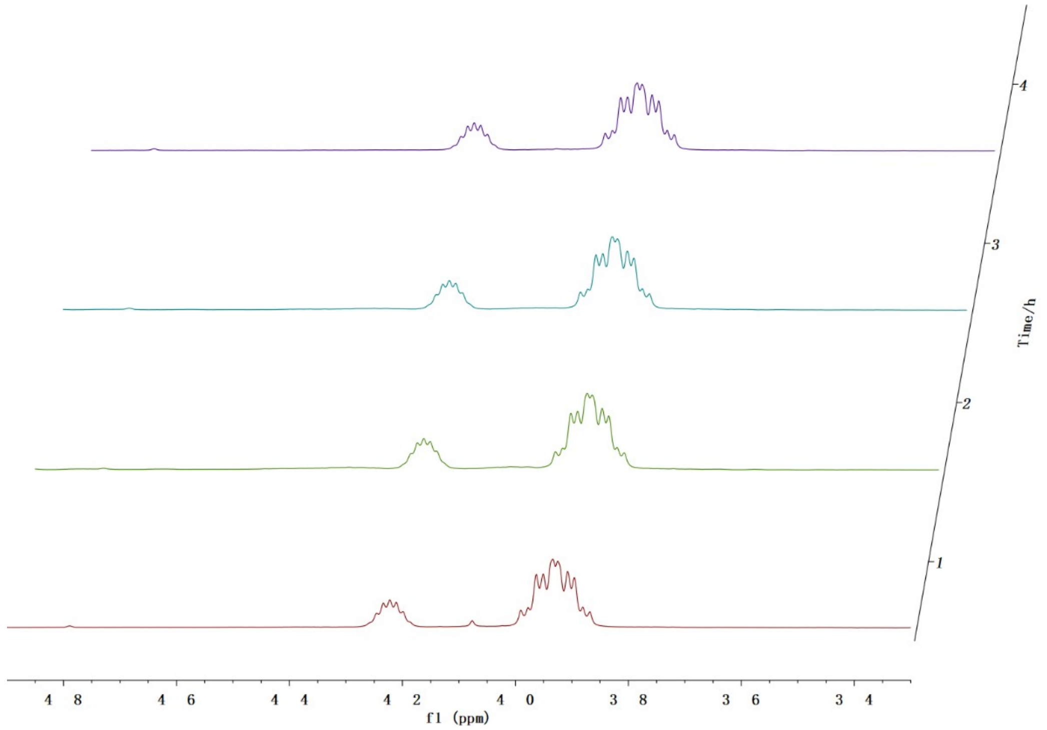

Fig. S30 The peaks of glycerin during the in situ monitoring in ^{1}H NMR with TPPO as internal standard.

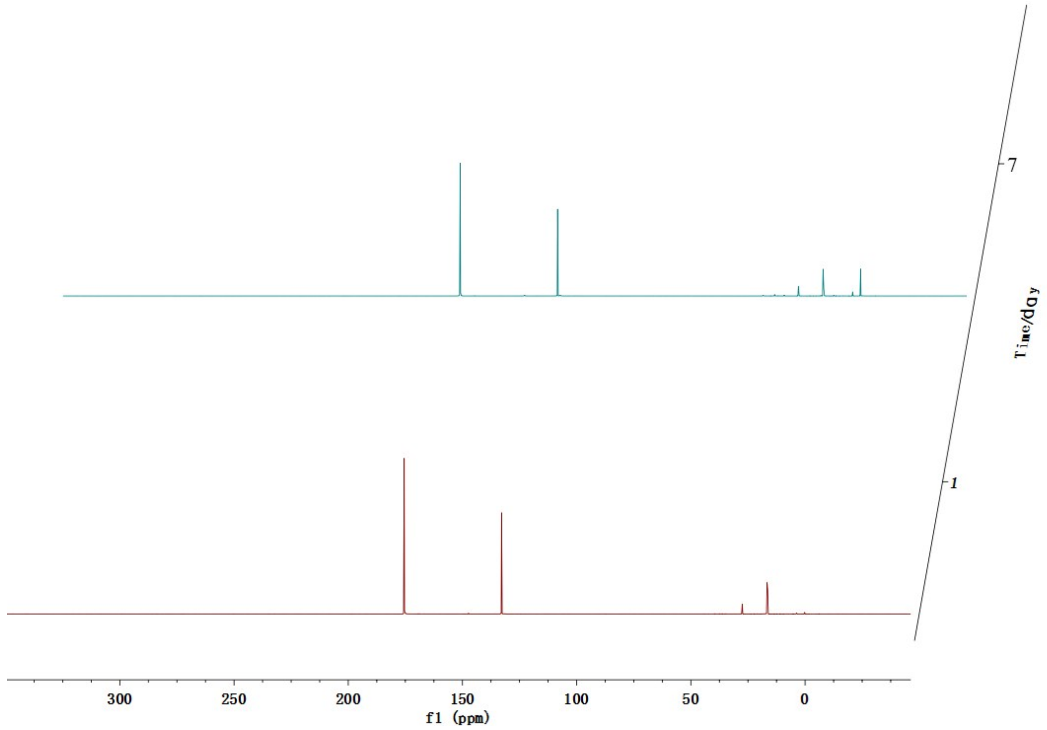

Fig. S31 Ex situ ^{31}P NMR monitoring for the sample added no model compound with TPPO as internal standard.

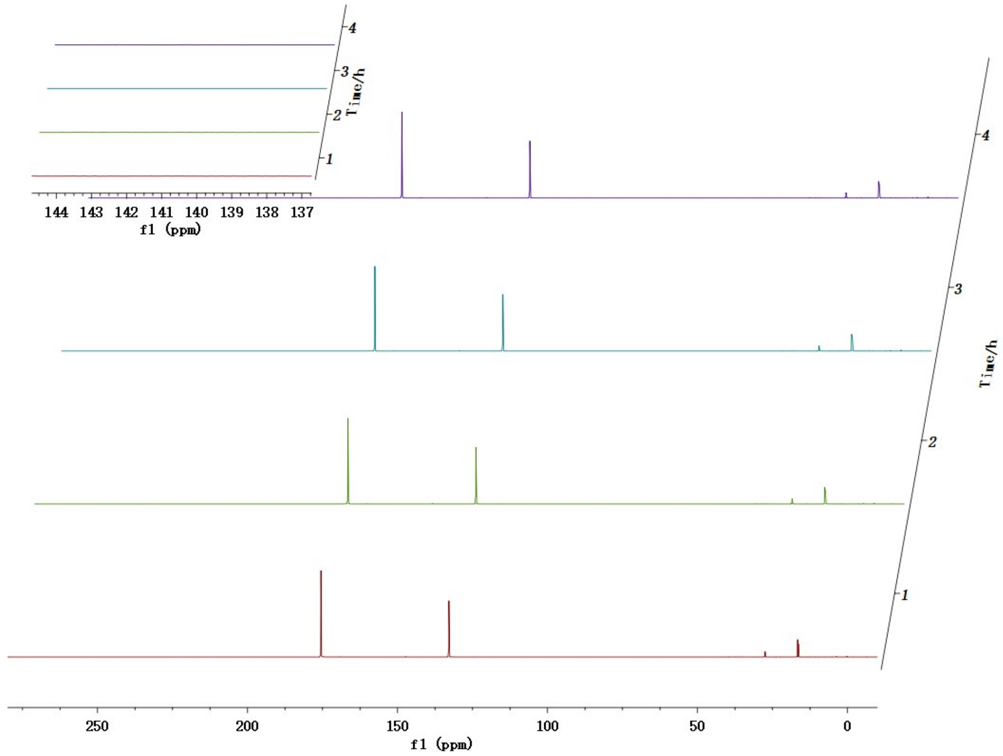

Fig. S32 In situ ^{31}P NMR monitoring for the sample added no model compound with TPPO as internal standard.

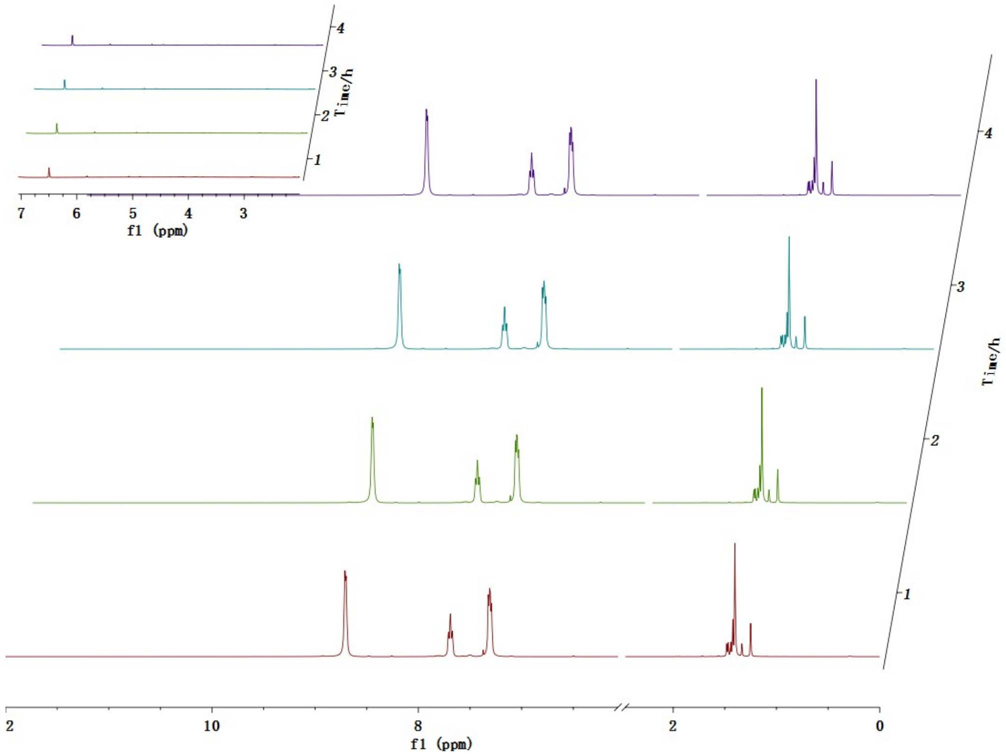

Fig. S33 In situ ^{1}H NMR monitoring for the sample added no model compound with TPPO as internal standard.

Table S1 Chemical shifts and integration regions for internal standards and functional groups for different hydroxyl groups (Reproduced from Ref. 32 with permission from the Royal Society of Chemistry. [a]

Functional group	Integration region (ppm)
Triphenylphosphine oxide (TPPO)	~27.5ppm
Aliphatic OH	150.0-145.5
Phenolic OH	144.7-137.3
Acid OH	136.6-133.6
Water peak	133.1-131.3<br>16.9-15.1

[a] chemical shift provided is for the corresponding hydroxyl group after derivatization with TMDP.

Table S2 The weights of different model compounds, ISTP solutions, anhydrous pyridine, and TMDP.

Sample	weight (mg)	weight of original solution and anhydrous pyridine (g)	weight of TMDP (g)
acetic acid	6.0	0.5891	0.1113
formic acid	1.0	0.5947	0.1148
benzoic acid	2.6	0.5962	0.1142
oxalic acid	2.7	0.5996	0.1152
methanol	1.1	0.5901	0.1145
1,2-Propylene glycol	2.4	0.5909	0.1129
glycerin	4.0	0.5933	0.1146
Ethylene glycol	5.5	0.5926	0.1144

Table S3 The test parameters of ^{31}P, ^{1}H and ^{13}C NMR.

Parameter	^{31}P NMR	^{1}H NMR	^{13}C NMR
TD	65536	65536	65536
SWH (Hz, ppm)	64102.6, 395.656	8012.82, 20.0234	26041.67, 258.779
AQ (s)	0.5111808	4.0894465	1.2582912
D1 (s)	25.000000000	8.000000000	1.500000000
NS	64	16	1500
RT	25. 511180800	12.089446500	2.758291200

Table S4 The content of TMDP and aliphatic OH and water wt% for the methanol during 4 hours of in situ stability tests with different D1.

Time	TMDP (mmol/g [a])		Aliphatic OH (mmol/g [a])		H_2O wt%[b]	
	D1=25s	D1=100s	D1=25s	D1=100s	D1=25s	D1=100s
1h	0.4346	0.4346	0.0351	0.0350	2.49	2.49
2h	0.4324	0.4325	0.0348	0.0348	2.55	2.54
3h	0.4319	0.4318	0.0346	0.0347	2.57	2.56
4h	0.4302	0.4302	0.0345	0..0345	2.58	2.58

[a] The calculation method: (Integration of spectral region of interest×mmol TPPO)/(Integration of TPPO region×total mass(g) of NMR sample)

[b] The percentage calculation method: (Integration of water region×mmol TPPO×278.29g/mol TPPO×100)/(1000×TPPO purity × Integration of TPPO region×total mass(g) of NMR sample)

Table S5 Naming of compounds mentioned in study.

Source of Compound	Chemical structure	Naming
Product derived from TMDP and formic acid	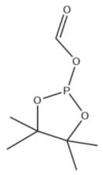	compound 1
Product derived from TMDP and water	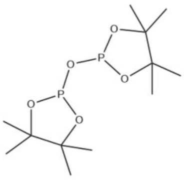	compound 2
One possible decomposition intermediate of compound 1	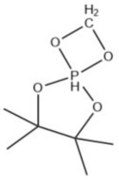	compound 3
One possible decomposition product of compound 1	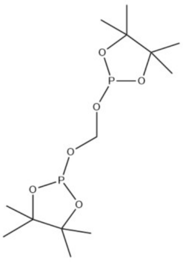	compound 4
Product derived from TMDP and acetic acid	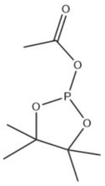	compound 5
One possible decomposition intermediate of compound 5	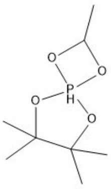	compound 6
One possible decomposition product of compound 5	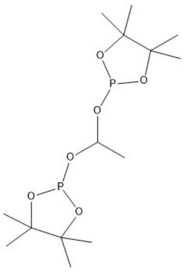	compound 7

Product derived from TMDP and oxalic acid	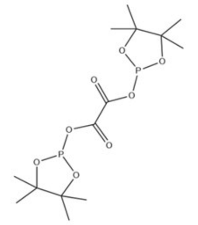	compound 8
Product derived from TMDP and benzoic acid	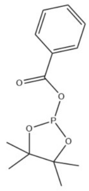	compound 9
Product derived from TMDP and methanol	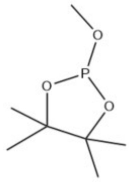	compound 10
Product derived from TMDP and ethylene glycol	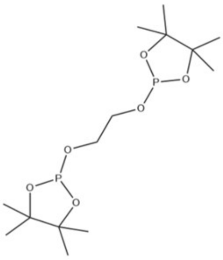	compound 11
Product derived from TMDP and 1,2-propanediol	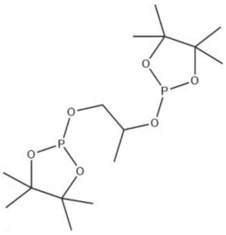	compound 12
Product derived from TMDP and glycerin	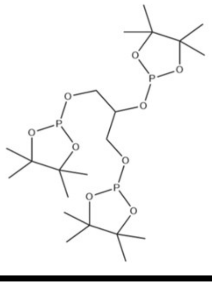	compound 13

Table S6 Different hydroxyl group contents and water wt% for the acetic acid during 4 hours of in situ and 14 days of ex situ stability tests with TPPO as internal standard.

Time	Aliphatic OH mmol/g [a]	Phenolic OH mmol/g [a]	Carboxylic OH mmol/g [a]	H_2O wt%[b]
1h	0.0007	0.0000	0.2415	3.95
2h	0.0005	0.0010	0.2395	4.00
3h	0.0015	0.0010	0.2376	3.99
4h	0.0027	0.0007	0.2361	4.03
1d	0.0079	0.0000	0.2177	4.17
7d	0.0241	0.0025	0.1767	2.39
14d	0.0345	0.0032	0.1474	1.53

[a] The calculation method: (Integration of spectral region of interest × mmol TPPO)/(Integration of TPPO region × total mass(g) of NMR sample)

[b] The percentage calculation method: (Integration of water region × mmol TPPO × 278.29g/mol TPPO × 100)/(1000 × TPPO purity × Integration of TPPO region × total mass(g) of NMR sample)

Table S7 Different hydroxyl group contents and water wt% for the benzoic acid during 4 hours of in situ and 14 days of ex situ stability tests with TPPO as internal standard.

Time	Aliphatic OH mmol/g [a]	Phenolic OH mmol/g [a]	Carboxylic OH mmol/g [a]	H_2O wt%[b]
1h	0.0000	0.0000	0.0220	1.95
2h	0.0002	0.0008	0.0216	1.94
3h	0.0005	0.0002	0.0213	1.98
4h	0.0000	0.0004	0.0209	1.99
1d	0.0005	0.0006	0.0204	2.10
7d	0.0005	0.0004	0.0200	2.73
14d	0.0014	0.0004	0.0184	2.84

[a] The calculation method: (Integration of spectral region of interest × mmol TPPO)/(Integration of TPPO region × total mass(g) of NMR sample)

[b] The percentage calculation method: (Integration of water region × mmol TPPO × 278.29g/mol TPPO × 100)/(1000 × TPPO purity × Integration of TPPO region × total mass(g) of NMR sample)

Table S8 Different hydroxyl group contents and water wt% for the methanol during 4 hours of in situ and 14 days of ex situ stability tests with TPPO as internal standard.

Time	Aliphatic OH mmol/g [a]	Phenolic OH mmol/g [a]	Carboxylic OH mmol/g [a]	H_2O wt%[b]
1h	0.0511	0.0000	0.0000	2.05
2h	0.0509	0.0004	0.0002	2.05
3h	0.0507	0.0004	0.0005	2.06
4h	0.0505	0.0004	0.0002	2.10
1d	0.0495	0.0000	0.0002	2.21
7d	0.0484	0.0000	0.0007	2.31
14d	0.0482	0.0006	0.0009	2.56

[a] The calculation method: (Integration of spectral region of interest × mmol TPPO)/(Integration of TPPO region × total mass(g) of NMR sample)

[b] The percentage calculation method: (Integration of water region × mmol TPPO × 278.29g/mol TPPO × 100)/(1000 × TPPO purity × Integration of TPPO region × total mass(g) of NMR sample)

Table S9 Different hydroxyl group contents and water wt% for the ethylene glycol glycol during 4 hours of in situ and 14 days of ex situ stability tests with TPPO as internal standard.

Time	Aliphatic OH mmol/g [a]	Phenolic OH mmol/g [a]	Carboxylic OH mmol/g [a]	H_2O wt%[b]
1h	0.1283	0.0000	0.0000	1.81
2h	0.1280	0.0009	0.0014	1.82
3h	0.1274	0.0013	0.0007	1.82
4h	0.1271	0.0008	0.0002	1.83
1d	0.1264	0.0007	0.0000	1.86
7d	0.1237	0.0007	0.0000	2.17
14d	0.1230	0.0007	0.0007	1.91

[a] The calculation method: (Integration of spectral region of interest × mmol TPPO)/(Integration of TPPO region × total mass(g) of NMR sample)

[b] The percentage calculation method: (Integration of water region × mmol TPPO × 278.29g/mol

TPPO×100)/(1000×TPPO purity × Integration of TPPO region×total mass(g) of NMR sample)

Table S10 Different hydroxyl group contents and water wt% for the 1,2 propanediol during 4 hours of in situ and 14 days of ex situ stability tests with TPPO as internal standard.

Time	Aliphatic OH mmol/g [a]	Phenolic OH mmol/g [a]	Carboxylic OH mmol/g [a]	H_2O wt%[b]
1h	0.0859	0.0000	0.0000	2.08
2h	0.0854	0.0000	0.0005	2.11
3h	0.0850	0.0000	0.0002	2.10
4h	0.0847	0.0004	0.0005	2.12
1d	0.0843	0.0004	0.0002	2.24
7d	0.0824	0.0007	0.0002	2.37
14d	0.0813	0.0004	0.0009	2.45

[a] The calculation method: (Integration of spectral region of interest×mmol TPPO)/(Integration of TPPO region×total mass(g) of NMR sample)

[b] The percentage calculation method: (Integration of water region×mmol TPPO×278.29g/mol TPPO×100)/(1000×TPPO purity × Integration of TPPO region×total mass(g) of NMR sample)

Table S11 Different hydroxyl group contents and water wt% for the glycerin during 4 hours of in situ and 14 days of ex situ stability tests with TPPO as internal standard.

Time	Aliphatic OH mmol/g [a]	Phenolic OH mmol/g [a]	Carboxylic OH mmol/g [a]	H_2O wt%[b]
1h	0.1770	0.0000	0.0000	2.02
2h	0.1754	0.0009	0.0007	2.04
3h	0.1746	0.0008	0.0008	2.05
4h	0.1737	0.0002	0.0005	2.07
1d	0.1714	0.0006	0.0008	2.11
7d	0.1703	0.0005	0.0002	2.13
14d	0.1696	0.0007	0.0003	2.12

[a] The calculation method: (Integration of spectral region of interest×mmol TPPO)/(Integration of

TPPO region×total mass(g) of NMR sample)

[b] The percentage calculation method: (Integration of water region×mmol TPPO×278.29g/mol TPPO×100)/(1000×TPPO purity × Integration of TPPO region×total mass(g) of NMR sample)

Table S12 The result of changes in the amount of phenolic OH in the sample added no model compound during in situ monitoring process.

Time/h	1	2	3	4
Phenolic OH mmol/g	0.0001	0.0003	0.0005	0.0001
